# *ENA1* deficiency attenuates *Saccharomyces ‘boulardii’* probiotic yeast virulence in immunosuppressed mouse fungaemia model

**DOI:** 10.1038/s42003-026-09763-z

**Published:** 2026-03-06

**Authors:** Alexandra Imre, Renátó Kovács, Ágnes Jakab, Andrea Harmath, Bálint Németh, Fruzsina Nagy, Lajos Forgács, Dávid Balázsi, László Majoros, Zsigmond Benkő, Nathan Crook, István Pócsi, Walter P. Pfliegler

**Affiliations:** 1 Department of Molecular Biotechnology and Microbiology, Faculty of Science and Technology, University of Debrecen, Debrecen, Hungary; 2 Department of Chemical and Biomolecular Engineering, North Carolina State University, Raleigh, NC USA; 3 Department of Medical Microbiology, Faculty of Medicine, University of Debrecen, Debrecen, Hungary; 4 Doctoral School of Pharmaceutical Sciences, University of Debrecen, Debrecen, Hungary; 5 Doctoral School of Nutrition and Food Sciences, University of Debrecen, Debrecen, Hungary

**Keywords:** Fungal genetics, Applied microbiology, Fungal infection

## Abstract

Recently, fungal infections originating from the probiotic *Saccharomyces ‘boulardii’* yeast are increasingly reported. Here, we aimed to reveal the background of and to diminish the virulence of this yeast, mitigating infection risks in vulnerable patient groups. Product and human isolates of *S. ‘boulardii’* were subjected to in-host selection and their subclone lineages were compared phenotypically to identify target phenotypes and associated genes. More virulent isolates showed signs of selection for high osmotic tolerance in immunosuppressed mouse model, hence the genes *NHA1* and *ENA1* were deleted in six different *‘boulardii’* backgrounds. Only *ENA1* deletion diminished virulence in our mouse fungemia model and it retained the ability for gut colonization and its probiotic characteristics, including similar effects on the gut microbiome in gavaged mice. We also demonstrated the successful substitution of the *ENA1* gene with an antilisterial bacteriocin, opening a strategy for safe strains with therapeutic effect. Our strain development approach highlighted the importance of testing various genetic backgrounds and resulted in engineered strains with drastically reduced capability to cause bloodstream infections even in immunosuppressed hosts, establishing the groundwork for safer probiotic yeast therapies in the future.

## Abstract (web summary)

A new probiotic *Saccharomyces ‘boulardii’* yeast strain with diminished virulence is developed using experimental infection and observations on in-host selection, achieved through the deletion of the *ENA1* osmotic stress gene.

## Introduction

The probiotic yeast, the so-called *‘boulardii’* subtype of *Saccharomyces cerevisiae*, clearly stands out in a market dominated by lactic acid-producing and other bacterial species as the most sought-after yeast probiotic globally^1,2^. Among others, it is used for the treatment of *Clostridioides difficile*-related and antibiotic-associated diarrhea^1^ and to improve the symptoms of irritable bowel syndrome (IBS)^3^. It is also researched for probiotic-enhanced food products and drinks^4^.

Despite the indisputable health benefits of *S.* *‘boulardii’*, there is a risk of fungemia among immunocompromised or severely ill patients, infants, and elderly people. In recent years, a growing number of such cases have been reported, often following the use or administration of yeast probiotic supplements^5–8^. The frequency of *Saccharomyces* probiotic fungemia cases is not known, and there is a lack reporting obligations, and so far, only a single retrospective analysis was published. This study suggested that, in the hospital examined, the incidence of fungemia cases for *S.* *‘boulardii’* was similar to that of *C. albicans*^8^. Scarcity of data is largely due to the lack of subtyping conducted during clinical diagnostics; thus, the number of undetected cases might greatly outnumber the published ones. Strain-level genotyping, e.g., with multiplex PCR, may ameliorate this shortfall in diagnostics^5^. In an assessment report from the European Medicines Agency^9^, a causal relationship was found between the use of *S.* *‘boulardii’* and fungemia. As a result, bloodstream infections have been indicated as a side effect of rare frequency on the package leaflet of yeast probiotic products in the European Union. Recently, a hospital in Belgium even completely discontinued the use of *S.* *‘boulardii’* probiotics based on safety concerns following high mortality rate in locally infected patients^6^. In our recent study, 48% of clinical isolates from the university hospital in Debrecen, Hungary, proved to be probiotic yeasts^10^.

At the same time, there is growing interest in extending the potential applications of *S.* *‘boulardii’* by the heterologous expression of various genes, and the introduction and modification of biosynthetic pathways for immune modulating^11^ and antimicrobial effects^12–16^ or other biotherapeutic applications^17,18^. The proposed applications of such genetically engineered probiotic yeast strains would often target already vulnerable patients who may have predisposing factors for fungal infections. Despite the obvious importance of safety concerns, there has been virtually no crosstalk between studies concerning the health benefits and genetic improvements of *S.* *‘boulardii’*, and those focused on its opportunistic pathogenicity and virulence determinants. Thus, an integrated approach putting both the patients and the yeast in focus is needed to better understand probiotic yeast infections, to improve their safety, and to extend their applicability.

On the patients’ side, knowledge has been steadily accumulating on *S.* *‘boulardii’* fungemia cases, which affect already hospitalized patients^6,8,19^. A total of 108 cases of *Saccharomyces* fungemia were recently analyzed in a systematic review, and they found that the most important risk factors were hospitalization at intensive care units, parenteral nutrition or enteral feeding, diarrhea, diabetes mellitus, immunosuppression, gastrointestinal surgery, and catheter use^20^.

Virulence factors of pathogenic yeast species, like *Candida* spp. or *Cryptococcus neoformans*, upon infection in humans are well-known^21^. However, the factors behind opportunistic pathogenicity concerning *S.* *“boulardii”* itself are less clear. In most cases, only a low number of product isolates have been compared^22,23^. In general, in vitro virulence factor tests failed to identify clear attributes enabling opportunistic pathogenicity in a mammalian model or in humans for *S.* *‘boulardii’* and in general, for *S. cerevisiae*^24^. Nevertheless, using mouse bloodstream infection models, the opportunistic pathogenic nature of the probiotic strain has been experimentally confirmed^25,26^. In one of our previous works, four commercial and ten clinical *S.* *‘boulardii’* isolates, the largest number of such isolates so far to be compared, were tested for their general phenotype, extracellular virulence factors, immune interactions, and virulence in *Galleria melonella* larva model^27^. No unequivocal difference was found that would indicate a specific trait that enables *S. ‘boulardii’* clinical isolates to acquire higher pathogenicity compared to the commercial strains, but the clinical isolates showed various phenotypic differences.

Since all previous genomics studies aiming to identify virulence factors in *Saccharomyces* put *S. cerevisiae* isolates and strains in the center of attention, the number of studies focusing on the genetic background of the probiotic yeast’s virulence attributes is scarce^28,29^. Our recent study confirmed that the loss of function of heme oxygenase-1 (*HMX1*), a known virulence factor in *Candida albicans*, elevated and not diminished virulence in six closely related yet distinguishable probiotic yeast genetic backgrounds in a mouse model^30^. These results highlighted the need for more thorough investigation on the opportunistic pathogenicity of *S. ‘boulardii’*, as the key factor in infections may not be active virulence factors but the ability of the yeast to survive the various stressful conditions it may encounter during a systemic infection^27,31^.

In the present study, we applied a new approach to uncover genetic factors enabling *S. ‘boulardii’* to cause fungemia and systemic infection. First the virulence of our probiotic yeast isolate collection was assessed by infection of immunosuppressed mouse model followed by kidney burden and lethality analysis. Founded on this data a selection of isolates was subjected to excessive spot plate stress phenotyping. Combined results of the data derived from mouse experiments and spot plate phenotyping led to the identification of stress resistance phenotypes and genetic traits being under in-host selection pressure. This approach helped us to target stress resistance genes, which were subsequently deleted, and the modified strains were assessed anew, along with whole genome analysis of each isolate and their knockout versions. With this method, we successfully engineered *S. ‘boulardii’* strains from six genetic backgrounds that all show greatly diminished kidney burden as well as up to 100% survival of infected mice. At the same time, gut colonization by a probiotic-derived knockout strain is shown to be comparable to a commercial isolate, with similar effects on fecal microbiota composition. We believe that such strains in the future may mitigate the risk of yeast probiotic treatments and may function as platform strains for development of designer probiotics with targeted therapeutic effects, combining safety and efficacy in a single yeast probiotic product. To illustrate this opportunity in next-generation yeast probiotics, we also show that the stress gene knockout probiotic yeast can be effectively modified to produce an anti-*Listeria* peptide without diminishing its broad antibacterial effect or growth in standard media.

## Results

### Selection regimen for wild type isolates, subclone lineages, and genetically modified strains for experiments

Yeasts in our collection are referred to as wild-type isolates instead of strains, as they were recently isolated from commercial products or from patients (Table 1), and their genetic stability has not been assessed. From these, so-called subclone lineages (single-cell-derived colonies) were isolated either after in vitro culturing or after re-isolation from mice. These may be referred to as in-vitro and in vivo-evolved lineages, but as the timeframe of our experiments was short (measured in days), we simply refer to them as in vivo or in vitro selected lineages. This implies selection forces acting upon standing and/or de novo genetic variation (i.e., clonal heterogeneity) found among the cell population of a single isolate. For the below described infection experiments, all four commercial and ten clinical isolates in our collection were used. For further analysis, six isolates were selected to represent both commercial (*n* = 2) and clinical (*n* = 4) samples, having higher and lower pathogenicity as well. Phenotyping experiments were carried out on ten in vitro selected and on 19−20 in vivo selected subclone lineages (from two different infected mice in all cases). Then the same six wild-type isolates were subjected to gene deletion and subsequent phenotyping and mouse infection. Finally, one probiotic isolate was chosen, based on its potential applicability as a biotherapeutic, to express an antibacterial peptide and to assess antibacterial activity before and after genetic modification. This probiotic isolate, and its gene-deleted and antibacterial peptide-producing derived strains, were subjected to further tests of applicability.

**Table 1:** Commercial and clinical *S. ‘boulardii’* isolates and patient data used in current study

Isolate Nos.	ID	Generated mutants	Type	Formulation	Species and strain composition of product	Place and date of aquisition			Country of manufacturing		References
**1**	PY0001	1: *ena1-Δ0/ena1-Δ0* 2: *nha1-Δ0/nha1-Δ0* 3: *ena1::LecC/ ena1::LecC*	commercial	active dry	*S. ‘boulardii’* CNCM I-745	Debrecen, Hungary, March 2015			France		^5,27,30,31^
**2**	PY0002	1: *ena1-Δ0/ena1-Δ0* 2: *nha1-Δ0/nha1-Δ0*	commercial	active dry	*S. ‘boulardii’* CNCM I-745	Debrecen, Hungary, Nov 2017			France		^5,27,30^
**3**	PY0003	–	commercial	active dry	*S ‘boulardii’* CNCM I-1079 + *R. rhamnosus* GG	Debrecen, Hungary, Sept 2017			Czech Republic		^5,27^
**4**	PY0004	–	commercial	active dry	*S. ‘boulardii’* CNCM I-1079 + *R. rhamnosus* GG	Debrecen, Hungary, Nov 2017			Czech Republic		^5,27^

Isolate Nos.	ID	Generated mutants	Type	Age (yr) at sampling	Sex	Medical condition during isolation	Mycosis case	Anatomical origin	Date of sampling	Geographic origin, unit	
**5**	DE3912	–	clinical	85	♂	pneumonia	no	trachea	31. Jan 2018	Debrecen, University Clinic, ICU	^5,27^
**6**	DE6507	1: *ena1-Δ0/ena1-Δ0* 2: *nha1-Δ0/nha1-Δ0*	clinical	63	♂	pneumonia	yes	haemoculture	18. Febr 2017	Debrecen, University Clinic, ICU	^5,27,30^
**7**	DE27020	–	clinical	40	♀	bacterial sepsis	no	bronchus	23. Aug 2015	Debrecen, University Clinic, ICU	^5,27,31^
**8**	DE35762	1: *ena1-Δ0/ena1-Δ0* 2: *nha1-Δ0/nha1-Δ0*	clinical	66	♀	respiratory failure	yes	haemoculture	5. Nov 2015	Debrecen, University Clinic, ICU	^5,27,30^
**9**	DE42533	–	clinical	2	♂	disorder of body-fluid homeostasis	no	throat	15. Dec 2017	Debrecen, University Clinic, inpatient care	^5,27^
**10**	DE42807	–	clinical	1	♀	diarrhea	no	vagina	4. Dec 2017	Debrecen, University Clinic, inpatient care	^5,27^
**11**	DE45866	–	clinical	64	♂	ischemic stroke	no	bronchus	29. Dec 2017	Debrecen, University Clinic, ICU	^5,27^
**12**	551/2018	–	clinical	81	♂	paralytic ileus	no	feces	3. Jan 2018	Szeged, University Clinic	^27^
**13**	465/2018	1: *ena1-Δ0/ena1-Δ0* 2: *nha1-Δ0/nha1-Δ0*	clinical	41	♀	amenorrhea	no	vagina	3. Jan 2018	Szeged, University Clinic	^27,30^
**14**	2251/2018	1: *ena1-Δ0/ena1-Δ0* 2: *nha1-Δ0/nha1-Δ0*	clinical	17	♂	ulcerative colitis	no	feces	8. Jan 2018	Szeged, University Clinic	^27,30^

### Clinical *S. ‘boulardii’* isolates show significant differences in virulence in mice

**Fig. 1:**
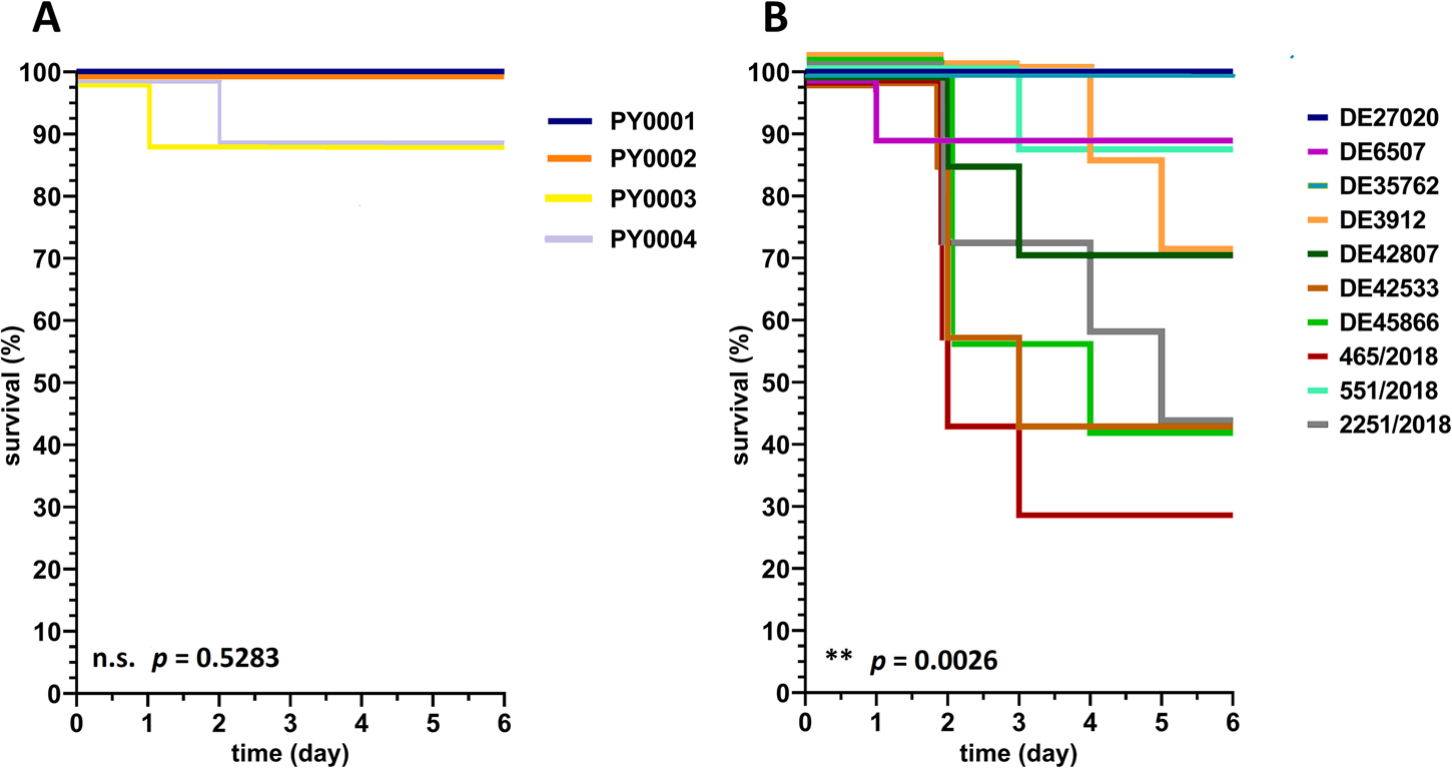
Kaplan-Meier survival curves of mice injected with probiotic yeasts. **A** Wild type commercial (*n* = 4) and (**B**) clinical (*n* = 10) *S. ‘boulardii’* isolates. Results of Log-rank (Mantel-Cox) test are shown in the bottom-left corner of the plots; significant differences between samples are shown (n.s.: non-significant; **: *p* < 0.01). The survival curves were slightly displaced when overlapping for clarity. Number of mice used for the experiments was *n* = 9 (PY0001, PY0002, PY0004, DE27020, DE6507, DE35762), *n* = 8 (PY0003, 551/2018), and *n* = 7 (DE3912, DE42807, DE42533, DE45866, 465/2018, 2251/2018).

We compared four products and ten clinical isolates of the probiotic yeast *S. ‘boulardii’* (Table 1) for their virulence in a mouse model. Infection experiments showed little to no difference (*p* > 0.05) in terms of virulence in case of the commercial isolates, but significant (*p* < 0.01) differences were found when clinical isolates were injected into the bloodstream of immunosuppressed mice (Supplementary Table 1 and Fig. 1). The following isolates showed remarkably high virulence: 465/2018 (28% mouse survival), DE45866, DE42533, and 2251/2018 (42% mouse survival) (Fig. 1).

**Fig. 2:**
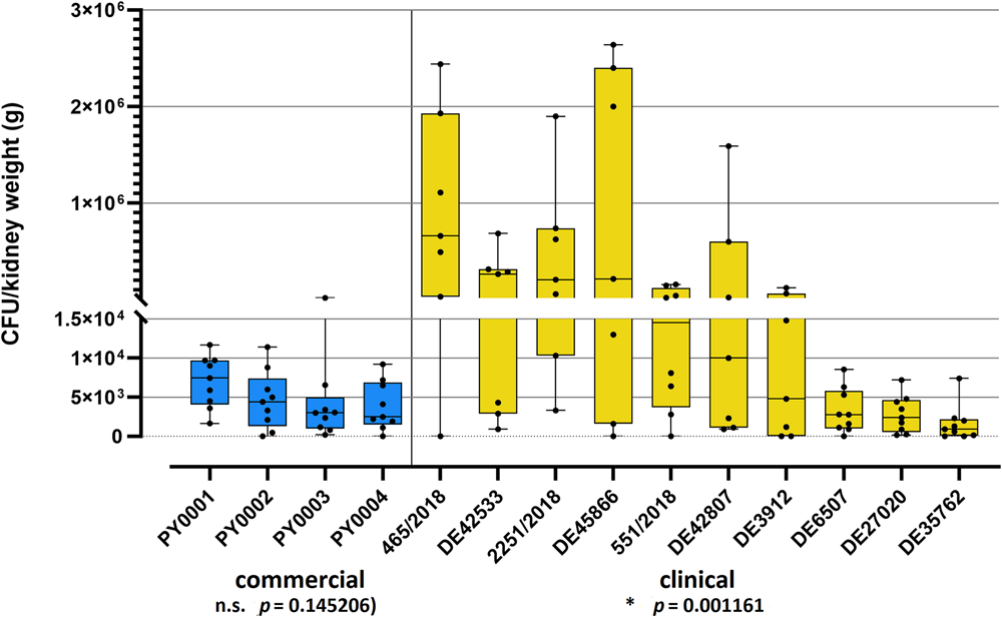
Fungal burden of kidneys in the case of commercial and clinical isolates of *S. ‘boulardii’.* An individual data point represents the kidney burden [CFU/kidney weight (g)] of an individual mouse. Data from mice that died during or were killed at the end of the experiment are both plotted. Horizontal black lines represent the median of the datapoints, whiskers extend to minimum and maximum values. Results of Kruskal–Wallis test with Bonferroni correction (*α* = 0.005) for the commercial and for the clinical isolates’ values are shown in bottom of the plot areas (n.s.: non-significant; *: *p* < 0.05). Number of mice used for the experiments was *n* = 9 (PY0001, PY0002, PY0004, DE27020, DE6507, DE35762), *n* = 8 (PY0003, 551/2018), and *n* = 7 (DE3912, DE42807, DE42533, DE45866, 465/2018, 2251/2018), as in Fig. 1.

Regarding kidney burden, the commercial isolates did not differ significantly (*p* > 0.05) from one another, while in the case of clinical isolates the CFU values of the isolate 2251/2018 were significantly higher compared to isolates DE27020, DE6507, and DE35762 [*p* < 0.01 (DE27020); *p* < 0.01 (DE6507); *p* < 0.01 (DE35762)] (Supplementary Table 1 and Fig. 1). The isolate 465/2018 also showed a remarkably high median CFU value (the highest one among the isolates) (Fig. 2).

### Subclones of virulent isolates show increased LiCl and NaCl tolerance

**Fig. 3:**
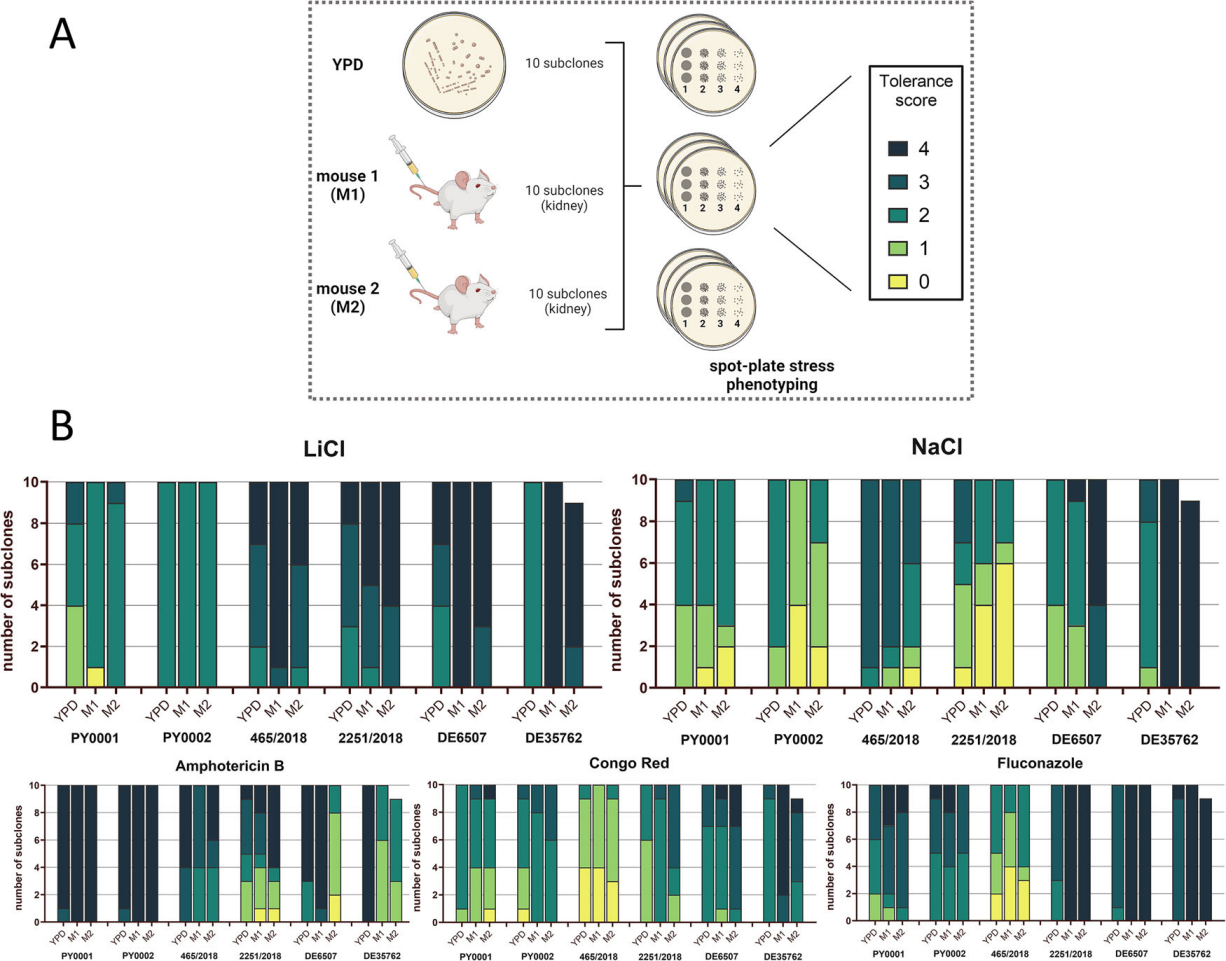
Spot-plate stress phenotyping results. **A** Schematic representation of the spot-plate stress phenotyping experiment conducted on subclone lineage. Panel A) was created in BioRender. Imre, A. (2026) https://BioRender.com/pbq57a0. **B** Compiled stress tolerance scores of in vitro and in vivo mouse kidney subclones derived from the commercial and clinical *S. ‘boulardii’* isolates. M1: mouse 1, M2: mouse 2. Tolerance scores are color-coded in stacked bar charts. Tolerance score 0: no visible growth; 1: growth of the first spot (containing 10^4^ cells); 2: growth of two spots (containing 10^4^ and 10^3^ cells); 3: growth of three spots (containing 10^4^, 10^3^ and 10^2^ cells); 4: growth of all 4 spots (containing 10^4^, 10^3^, 10^2^ and 10 cells). The number of individual subclone lineages was *n* = 10 for each experiment, except for DE35762 mouse 2 (*n* = 9).

To reveal whether virulence and increased survival is associated with differences in stress tolerance, spot plate stress phenotyping was applied for a set of *S. ‘boulardii’* wild-type isolates, that were chosen based on their virulence in mice (Fig. 3a). Hence, PY0001 and PY0002 (all inoculated mice survived) and 465/2018 as well as 2251/2018 (elevated virulence) were chosen for this test. The two patient hemoculture isolates, DE6507 and DE35762, were also included. In case of all six isolates, ten in vitro subclones were isolated from YPD broth, named as YPD-SubClones (YPD-SCs). Ten in vivo subclones each from the kidneys of two BALB/c mice (Mouse1: M1 and Mouse2: M2) were isolated to account for clonal heterogeneity in a single strain (Fig. 3a), a common phenomenon especially in non-haploid microbes as the probiotic yeast. These were named as Mouse-SubClones (M-SCs). Based on the stress tolerance score, we determined whether the growth of the subclones was weak (score 1), medium (score 2 and 3), or strong (score 4). Subclones with tolerance score 0 were considered non-viable under the given stress.

A marked difference in LiCl tolerance was observed between commercial and clinical subclones (Fig. 3b). While growth was generally weak or medium in the case of in vitro and mouse subclones of PY0001 and PY0002 commercial isolates, a significant number of in vitro and mouse subclones of clinical isolates showed strong tolerance against LiCl. Additionally, mouse subclones of these clinical isolates always showed even higher LiCl tolerance than the in vitro subclones of the same isolate (Fig. 3b).

In the case of PY0001, 40% of in vitro subclones showed weak growth on NaCl plates, while this ratio was 35% percent for mouse subclones. Some mouse subclones could not grow under this stress condition (Fig. 3b). All in vitro subclones and 85% of mouse subclones in case of 465/2018 showed medium growth, thus their NaCl tolerance was higher than the subclones of commercial isolate subclones (Fig. 3b). In contrast 40% of the in vitro subclones of 2251/2018 showed weak growth, and 10% of the subclones did not grow. In the case of mouse subclones of the same isolate, 50% did not grow, and 15% grew weakly. Remarkably, 35% of the mouse subclones of DE6507 and 100% of the mouse subclones of DE35762 showed strong growth, which was not observed for the subclones of other isolates when NaCl stress was applied (Fig. 3b).

Amphotericin B tolerance was generally high among commercial isolates and their mouse subclones compared to clinical isolates and mouse subclones (Fig. 3b). 90%–100% of the commercial in vitro and mouse subclones showed strong growth in the presence of amphotericin B, while this ratio was lower in case of 465/2018 (YPD-SCs: 60%, M-SCs: 20%), 2251/2018 (YPD-SCs: 10%, M-SCs: 40%), and DE6507 (YPD-SCs: 70%, M-SCs: 45%). All in vitro subclones of DE35762 showed strong growth, however mouse subclones of the same isolate had much lower tolerance (nearly 50% of the subclones were barely viable on the plates) (Fig. 3b).

Tolerance against Congo Red was isolate-dependent. However, the subclones of 465/2018 were remarkably sensitive, since 90% of in vitro subclones and 95% of mouse subclones showed no or weak growth (Fig. 3b).

In case of fluconazole, majority of the commercial in vitro and mouse subclones of PY0001 showed weak (YPD-SCs: 20%, M-SCs: 5%) or medium growth (YPD-SCs: 80%, M-SCs: 70%). 25% of the mouse subclones showed strong growth (Fig. 3b). 90% of both PY0002 in vitro and mouse subclones showed medium growth. A high number of 465/2018 subclones grew weakly on fluconazole (YPD-SCs: 50%, M-SCs: 60%), meaning that these subclones were sensitive to fluconazole (Fig. 3b). Interestingly, all the in vitro subclones of 2251/2018, DE6507, and DE35762 showed medium growth, while all the mouse subclones of these isolates showed strong growth (Fig. 3b).

### Knocking out *ENA1* and *NHA1* as potential pathogenicity determinant genes

The *ENA1* gene encodes a P-type ATPase, which maintains the ionic balance between the two sides of the cell membrane. In our *S. ‘boulardii’* isolate, PY0001, it is located on chromosome IV from base 526602 to base 529877, as a single copy gene in contrast to the S288c reference genome, where it is found in three tandem copies^30^. This membrane protein allows yeast to survive under high salinity or alkaline conditions. It is also capable of exporting Li^+^ and K^+^ ions at low salt concentrations. The baseline expression of the *ENA1* gene is low, but it is rapidly induced by stress (osmotic, saline, alkaline)^32^. As a Na^+^/H^+^ antiporter the *NHA1* gene is responsible for the sodium and potassium efflux at acidic pH^33,34^, and it is also a sing-copy gene in PY0001^30^. Interestingly, the *ENA1* gene has been identified as a virulence gene in case of the pathogenic Basidiomycota yeast *Cryptococcus neoformans*^35^. However, it is important to emphasize that the regulation of *ENA1* and sensitivity to monovalent cations due to the deletion of this gene remarkably differ in *C. neoformans* and *Saccharomyces*^35^. In the former, deletion mutants exhibited diminished tolerance of alkaline, but not salt stress.

Since the function of *ENA1* and *NHA1* might contribute to the high NaCl and LiCl tolerance (in *Saccharomyces*) that was observed in the case of our clinical isolates and their subclones, our hypothesis was that *ENA1* and/or *NHA1* are important genes for virulence, helping the colonization and pathogenicity of *S. ‘boulardii’*. To test this hypothesis, we decided to delete these genes in the strains PY0001, PY0002, 465/2018, 2251/2018, DE6507, and DE35762, leading to the generation of 12 knock-out strains (Table 1.).

To investigate the importance of *NHA1* and *ENA1* in the virulence of our isolates, the genes were deleted in a marker-less fashion in six chosen isolates: two commercial isolates (PY0001, PY0002), two isolates that showed high virulence in immunosuppressed mice (465/2018, 2251/2018), and two isolates that originated from human bloodstream (DE6507, DE5762) using the CRISPR/Cas9 technology. Deletion of the genes was verified by PCR targeting the loci and also by sequencing and analyzing the whole genomes of the deletion strains. Compared to their respective wild-type isolates, deletion strains did not show marked alterations in ploidy, genome structure, mitochondrial DNA, or heterozygosity (Supplementary Tables 2–4 and Supplementary Fig. 1a–f). A single large, segmental duplication on the non-targeted chr. XVI and a deletion on chr. V was observed in DE35762 *nha1-Δ0/nha1-Δ0*. Furthermore, a 33,570 bp deletion was observed on the chr. IV of PY0001 *nha1-Δ0/nha1-Δ0*, DE6507 *nha1-Δ0/nha1-Δ0*, and DE6507 *ena1-Δ0/ena1-Δ0* in a region not targeted by our gene deletion approach (Supplementary Table 2, Supplementary Fig. 1a, e, f). The targeted *ENA1* and *NHA1* genes were all deleted starting and ending at the exact same loci on the respective chromosomes (Supplementary Table 2 and Supplementary Fig. 2).

Heterozygosity was low in all genomes, ranging from 2336 to 2750 heterozygous positions (thereby not exceeding 0.025% of the whole nuclear genome). These heterozygosities were consistently distributed primarily on chr. I, II, III, the left arm of IV, X, XII, XIII, and XVI. In *NHA1* deletion strains, 95.4% to 98.3% of alleles, and in *ENA1* deletion strains, 98.3 to 99.9% of alleles, remained heterozygous compared to the original wild type (Supplementary Table 3 and Supplementary Fig. 1a–f). Mitochondrial DNA copy number variation was minor, overall calculated copy numbers ranged between ~16.4 and 26.8 per haploid genome in all genomes, and the naturally occurring 2 µ plasmid of the yeast was present in all but one cases in higher copy numbers in knockout strains than in the wild types, their copy numbers ranging from 15.4 to 43.5 among the genomes (Supplementary Table 4).

**Fig. 4:**
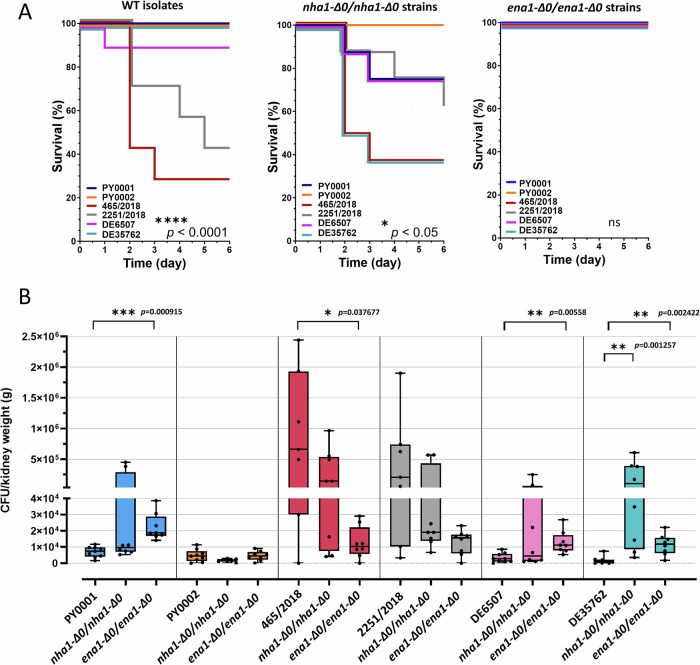
Virulence exhibited by the deletion strains in six-day-long infection experiments. **A** Kaplan-Meier survival curves of immunosuppressed BALB/c mice in 6-day-long infection experiments. The plot shows the same dataset for the 6 chosen isolates as it was depicted on Fig. 1. Results of Log-rank (Mantel-Cox) test are shown in the down-right corner of the plots. The survival curves are slightly displaced when overlapping for clarity. **B** Kidney burden from the same mice as in (**A**). An individual data point represents the kidney burden [CFU/kidney weight (g)] of an individual mouse. Data from mice that died during or were killed at the end of the experiment are both plotted. Horizontal black lines represent the median of the data points. Whiskers extend to minimum and maximum values and individual data points are shown. Significant differences between isolates and respective deletion strains are shown as follows: *: *p* < 0.05; **: *p* < 0.01; ***: *p* < 0.001; ****: *p* < 0.0001. Number of mice used for the experiments was 9 (PY0001, PY0002, DE6507, DE35762), 8 (PY0001 *nha1-Δ0/nha1-Δ0*, PY0001 *ena1-Δ0/ena1-Δ0*, *PY0002 ena1-Δ0/ena1-Δ0*, 465/2018 *nha1-Δ0/nha1-Δ0*, 465/2018 *ena1-Δ0/ena1-Δ0*, 2251/2018 *nha1-Δ0/nha1-Δ0*, 2251/2018 *ena1-Δ0/ena1-Δ0*, DE6507 *nha1-Δ0/nha1-Δ0*, DE6507 *ena1-Δ0/ena1-Δ0*, DE35762 *nha1-Δ0/nha1-Δ0*, DE35762 *ena1-Δ0/ena1-Δ0*), and 7 (PY0002 *nha1-Δ0/nha1-Δ0*, 465/2018, 2251/2018).

Surprisingly, deletion of *ENA1* but not *NHA1* diminished virulence in *S. ‘boulardii’*. The verified deletion strains were injected into immunosuppressed mice, and we followed the survival of the animals and determined the kidney burden in a 6-day-long infection experiment. Mouse survival of *NHA1* deletion mutants was strain-dependent and varied between 35 and 100% (Fig. 4a). However, in the case of *ENA1* deletion strains, mouse survival was 100% regardless of whether the strains were derived from commercial or clinical yeast isolates (Fig. 4a). Similarly to the wild type isolates, these strains were also re-isolated from the kidneys. The median of the CFU values from individual mouse kidneys for the *NHA1* deletion mutants increased with the virulence of the strain, thus in case of the avirulent PY0001 *ena1-Δ0/ena1-Δ0* it was 1.5 × 10^3^ CFU/g, in case of the moderately virulent PY0001 *nha1-Δ0/nha1-Δ0*, 2251/2018 *nha1-Δ0/nha1-Δ0* and DE6507 *nha1-Δ0/nha1-Δ0* strains it was 9.7 × 10^3^ CFU/g, 1.9 × 10^4^ CFU/g and 4.4 × 10^3^ CFU/g, respectively. In the case of the most virulent strains, 465/2018 *nha1-Δ0/nha1-Δ0* and DE35762 *nha1-Δ0/nha1-Δ0* it was an order of magnitude higher, 1.4 × 10^5^ CFU/g and 1.0 × 10^5^ CFU/g, respectively (Fig. 4b and Supplementary Table 5). On the contrary, median CFU values of the *ENA1* deletion mutants were similar across the strains: 1.9 × 10^4^ CFU/g (PY0001 *ena1-Δ0/ena1-Δ0*), 4.7 × 10^3^ CFU/g (PY0002 *ena1-Δ0/ena1-Δ0*), 1.0 × 10^4^ CFU/g (465/2018ΔΔ *ena1-Δ0/ena1-Δ0*), 1.6 × 10^4^ CFU/g (2251ΔΔ *ena1-Δ0/ena1-Δ0*), 1.1 × 10^4^ CFU/g (DE6507ΔΔ *ena1-Δ0/ena1-Δ0*), and 1.2 × 10^4^ CFU/g (DE35762 *ena1-Δ0/ena1-Δ0*) (Fig. 4b and Supplementary Table 5).

**Fig. 5:**
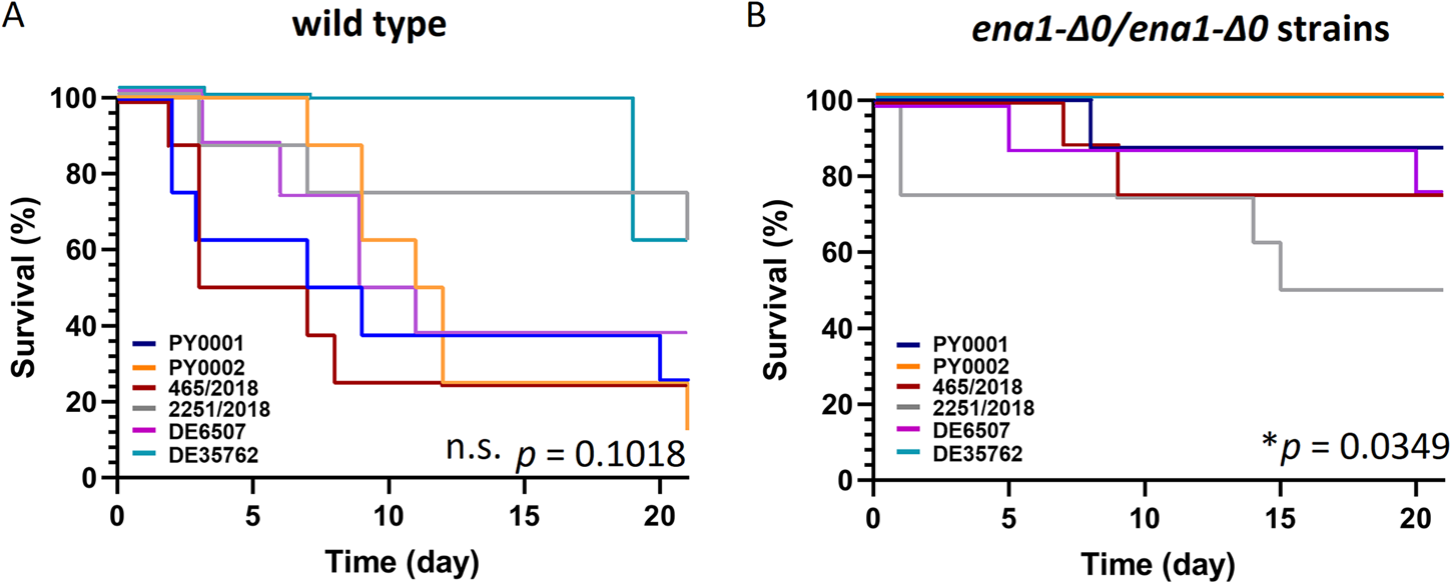
21-day survival tests of immunosuppressed mice infected with wild-type and deletion strains. **A** Wild-type isolates. **B** Respective *ENA1* deletion strains. Kaplan-Meier survival curves and results of Log-rank (Mantel-Cox) test are shown, survival curves are slightly displaced when overlapping for clarity. Differences are shown as follows: n.s.: not significant; *: *p* < 0.05. Number of mice used for the experiments was *n* = 8 for each yeast.

To further assess virulence, another 21-day-long infection experiment of immunosuppressed mice was conducted. As only *ENA1* deletion mutants and not *NHA1* deletion mutants showed promising results, the latter were omitted from this experiment. In this round of experiment, bloodstream infection with wild-type isolates resulted in 12–62.5% survival while *ENA1* deletion mutants showed values between 50 and 100% (Fig. 5 and Supplementary Table 6). In the case of PY0001, PY0002, and 465/2018 the deletion mutants showed significantly higher mouse survival than the wild type isolates. In the case of PY0001, the overall 25% survival of mice in the 21 days changed to 87.5% in the deletion mutant. In the case of PY0002, the 12.5% survival of the wild type changed to 100% in the *ENA1* deletion strain. The wild-type 465/2018 isolate’s 25% survival was elevated to 75% upon *ENA1* deletion. Survival curve differences were non-significant in the case of 2251/2018, DE6507, and DE35762 [pairwise Log-rank (Mantel-Cox) tests].

### The reassessment of stress tolerance in *ENA1* deletion strains revealed variations across different genetic backgrounds

**Fig. 6:**
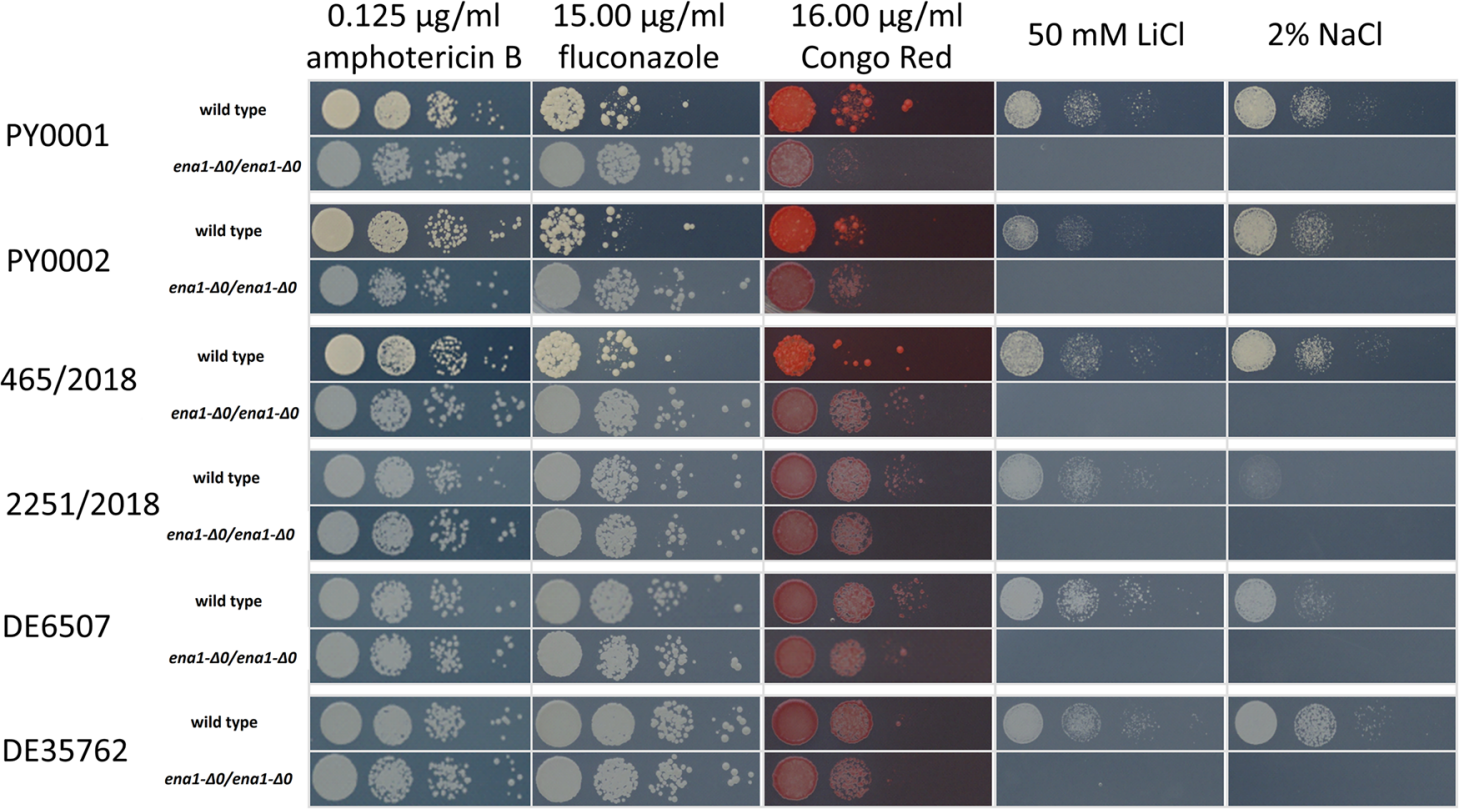
Spot-plate stress phenotyping results of wild type isolates and their *ENA1* knock-out counterparts. Inoculated spots on photographed stress agar plates contained 10^4^, 10^3^, 10^2^, and 10 cells, from left to right. Stress conditions are described on the top of the image.

Based on the mouse survival and kidney burden data, we focused on *ENA1* deletion strains as their pathogenic potential was greatly diminished. The knockout of *ENA1* did not affect amphotericin B tolerance but resulted in higher tolerance in the case of PY0001, PY0002, and 465/2018 when fluconazole was applied on agar medium. Change in Congo red tolerance was strain dependent (Fig. 6). In the case of PY0002, DE6507, and DE35762, tolerance did not change, in the case of PY0001 and 2251/2018, the tolerance decreased, and in the case of 465/2018 tolerance increased. All the *ENA1* deletion strains were non-viable on LiCl and NaCl plates with the same stressor concentrations that were applied previously in the case of the wild-type isolates (Fig. 6).

### Probiotic and biotherapeutic potential of *ENA1* deletion strains

Once the diminished pathogenicity of *ENA1* deletion mutants was established in various genetic backgrounds, we assessed whether knockout versions of the product isolate PY0001 can be cultured in standard media or show defective growth, potentially hindering their applicability as probiotics. Using small-scale laboratory tests, we showed that the knockout strain did not show significant difference in growth on standard yeast media, or in liquid cultures, and had identical doubling times and biomass production (Supplementary Figs. 4–5 and Supplementary Tables 7–9). Based on these favorable results, *ENA1* was not merely deleted but also successfully substituted in PY0001 with a synthetic Leucocin C bacteriocin-coding construct (Supplementary Fig. 2). The resulting recombinant PY0001 *ena1::LecC/ena1::LecC* strain displayed complete survival of infected mice in the 21-day-long experimental infection (Supplementary Table 6, Supplementary Fig. 3) and also showed similar growth as the wild type or the PY0001 *ena1-Δ0/ena1-Δ0* (Supplementary Figs. 4, 5, and Supplementary Tables 7–9). Experiments on liophilization survival showed PY0001 *ena1::LecC/ena1::LecC* to have the highest values, followed by PY0001, and PY0001 *ena1-Δ0/ena1-Δ0* under small-scale laboratory conditions (Supplementary Table 10). PY0001 *ena1::LecC/ena1::LecC* was also subjected to comparative genomic analysis as described above and was shown to have a trisomy of chr. I (Supplementary Fig. 1a). A draft assembly confirmed the correct integration of the LecC gene with a 5’ α-mating factor secretion signal sequence at the locus of the target *ENA1* (Supplementary Fig. 2c). Heterozygosity in this strain increased to 102.8% compared to the original PY0001 isolate (Supplementary Fig. 1a and Supplementary Table 3).

Regarding the tolerance of higher pH values that characterize the human gastrointestinal system, where probiotics are mostly expected to colonize, PY0001 *ena1-Δ0/ena1-Δ0* and PY0001 *ena1::LecC/ena1::LecC* were tested on buffered media for their growth. At pH values representing those found in the duodenum, middle and distal small intestine, and cecum (pH 6.1, 7.1, 7.5 and 6.0, respectively), both the PY0001 probiotic isolate and the two deletion strains showed high tolerance, while at a pronounced alkali stress of pH 8.0 the deletion strains showed markedly decreased tolerance (Supplementary Fig. 6 and Supplementary Table 11). Tolerance of bile acids was also shown to be similar in PY0001, PY0001 *ena1-Δ0/ena1-Δ0*, and PY0001 *ena1::LecC/ena1::LecC* (Supplementary Fig. 6 and Supplementary Table 11). We also determined MIC values for planctonic cells in liquid medium for commonly used antimycotics for these three yeasts, as a substantially increased tolerance of antifungals would be undesirable in probiotic strains. For Amphotericin B, the MIC values was diminished in PY0001 *ena1::LecC/ena1::LecC* (1 mg/L to 0.5 mg/L), and for fluconazole, both PY0001 *ena1-Δ0/ena1-Δ0* and *ena1::LecC/ena1::LecC* showed diminished values (2 mg/L to 0.5 mg/L). However, the two deletion strains displayed increased MIC values for caspofungin (0.004 mg/L to 0.06 and 0.03 mg/L) and micafungin (0.5 mg/L to 1 mg/L) (Supplementary Table 12).

**Fig. 7:**
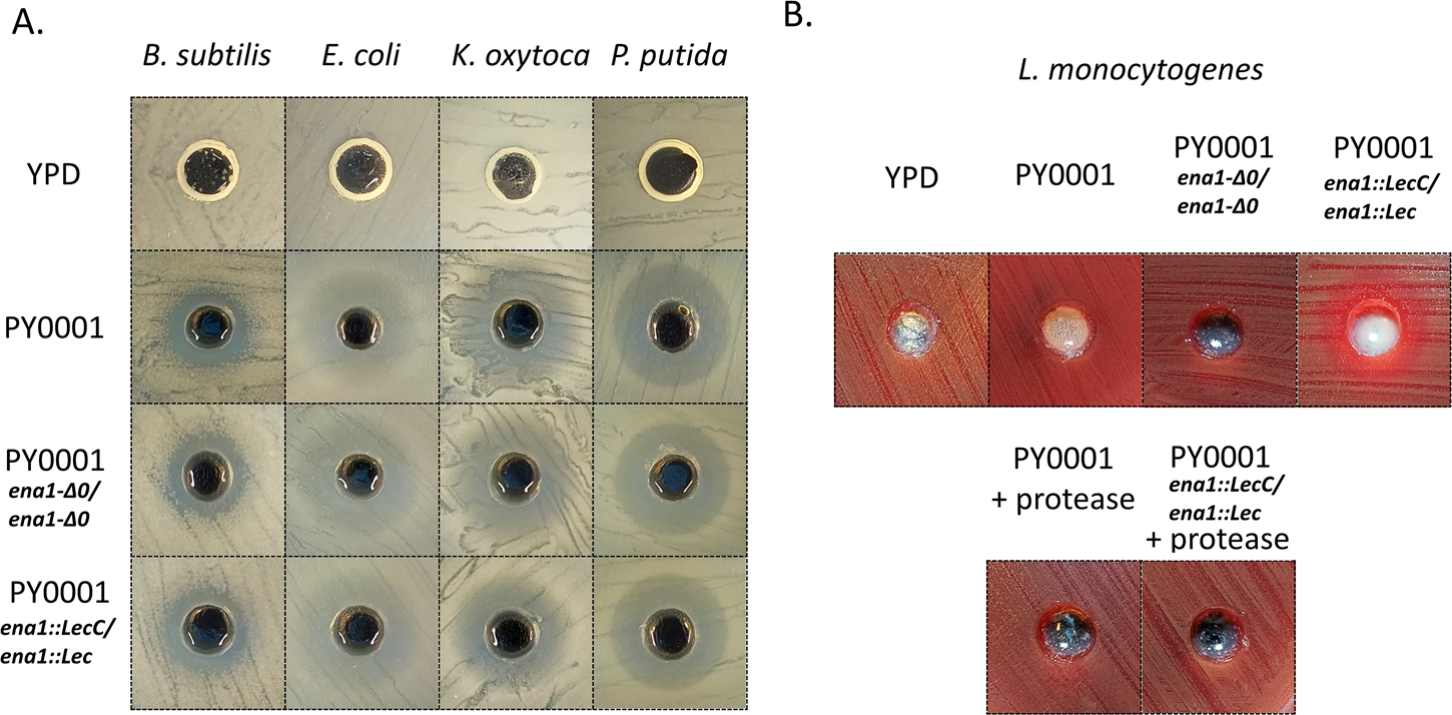
Antagonism test results with probiotic yeasts. **A** Inhibitory effect of the culture supernatants of the wild type PY0001 and its modified strains, PY0001 *ena1-Δ0/ena1-Δ0* and PY0001 *ena1::LecC/ena1::LecC* against four bacterial species on Nutrient Broth agar. As a control, sterile YPD medium was used. **B** Specific inhibitory effect of the concentrated dialysed supernatant of PY0001 *ena1::LecC/ena1::LecC* against *L. monocytogenes* on Mueller-Hinton agar. Measurements and statistical comparison are described in Supplementary Table 13. Number of replicates for antagonism tests was *n* = 3 (technical replicates).

To test antibacterial activity of the PY0001 isolate and its two modified versions, we assessed the inhibitory effect of the culture supernatants of the yeasts cultured in YPD liquid medium. Supernatants were in the pH range of 4.25–4.30. All three tested yeast supernatants could inhibit the growth of the tested *E. coli*, *B. subtilis*, *K. oxytoca*, and *P. putida* strains in a similar manner, without significant differences (Fig. 7 and Supplementary Table 13). The antibacterial activity of the supernatants was lost when the pH was set to 6.0 before tests. The ability to express and secrete the bacteriocin by the PY0001 *ena1::LecC/ena1::LecC* strain was verified by the inhibitory effect of its concentrated and dialysed supernatant against *L. monocytogenes*. This effect was completely abolished by protease treatment of the dialysate, and in this case, PY0001 and PY0001 *ena1-Δ0/ena1-Δ0* did not show any inhibition against this bacterium (regardless of the pH of the supernatant), verifying that the bacteriocin production was successfully achieved in PY0001 *ena1::LecC/ena1::LecC*.

### Avirulent *S. ‘boulardii’* strain (PY0001 *ena1-Δ0/ena1-Δ0*) showed comparable gut viability to the original commercial *S. ‘boulardii’* (PY0001)

**Fig. 8:**
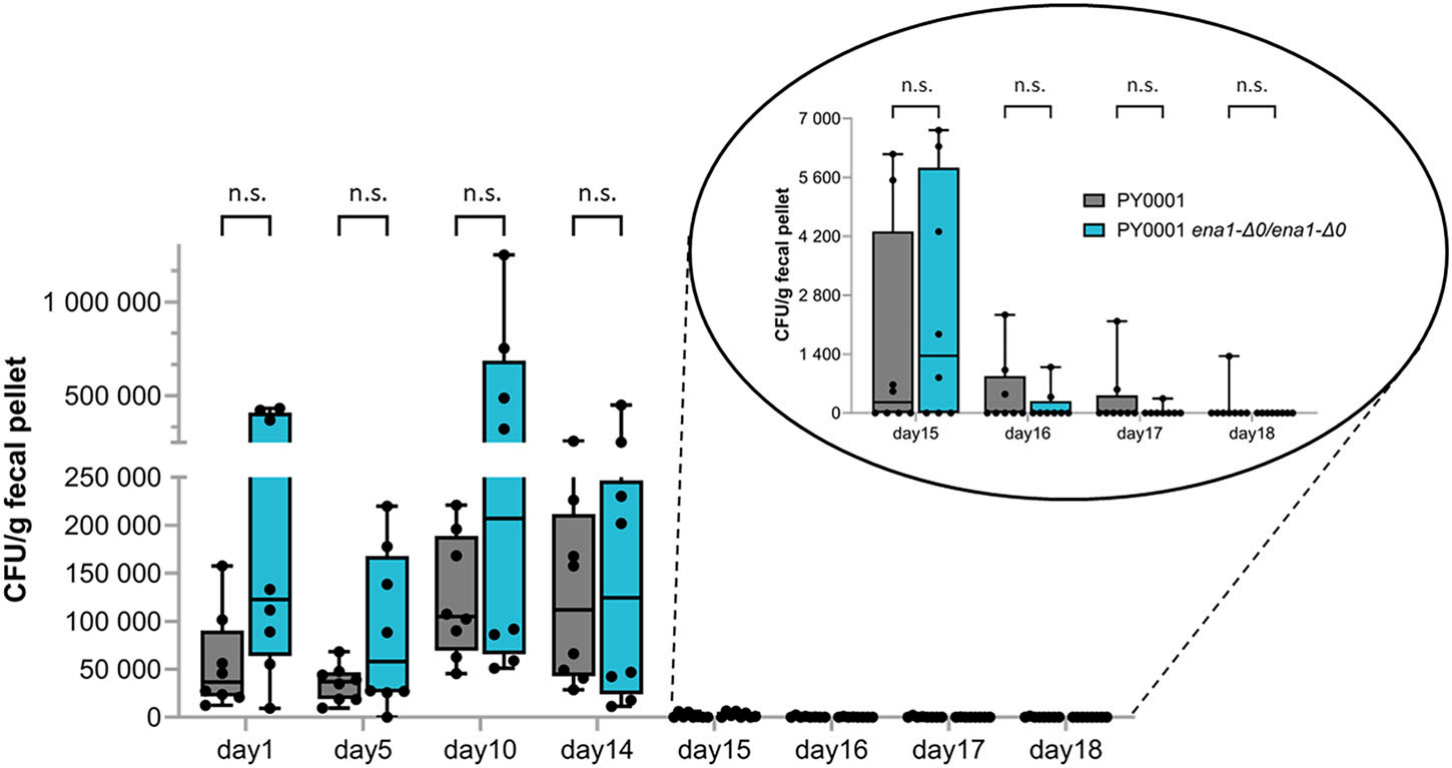
Colonization of the mouse gut during gavaging. Yeast CFU/g levels in fecal pellets during and after gavaging mice for two weeks are shown. The inset shows the rapidly declining density of yeasts after gavaging was stopped (days 15–18). n.s.: non-significant. Box plots show individual data, median value as horizontal lines, and whiskers show minimum and maximum values. Gavaging was started on day 0 and finished on day 13. Number of mice used for the experiments was *n* = 8 in each of the three groups.

Since the *ENA1* knock-out strain of PY0001 showed drastically diminished virulence in our mouse fungemia model, this knockout, the PY0001 *ena1::LecC/ena1::LecC* strain, and their parental strain (PY0001) were chosen to test and compare viability in the gastrointestinal tract. First, in vitro assessment showed that the survival under simulated gastrointestinal conditions was not changed in the knockout strains compared to PY0001 (Supplementary Table 14). Based on these favorable results, PY0001 and the PY0001 *ena1-Δ0/ena1-Δ0* knockout strains were tested for their ability to colonize the GI tract in C57BL/6 J mice. We showed that all mice gavaged for 14 days survived, and mouse weight was statistically not different in the two yeast-gavaged groups compared to the control group gavaged with water (Supplementary Figs. 7 and 8). Colony forming unit was determined on day 1, day 5, day 10, and day 14–18 by plating suspended fecal pellets on DRBC agar plates described above. No colonies were observed in case of the control group gavaged with water. In case of the groups that were gavaged with yeast, there was no significant difference in CFU values of the two yeast strains on days 1 (day after first gavaging), 5, 10, and 14 (day after last gavaging) when sampling was performed (Fig. 8). Clearance between day 15 and day 18 was similar as well, since CFU values did not show significant difference between the two yeasts (Fig. 8). The gavaged yeasts reached ~9.64 × 10^3^ to ~2.59 × 10^5^ CFU/g cell density in the case of PY0001 during the gavaging experiment (days 1–14, the day after the first gavaging and after the last, respectively) and 0 to ~1.25 × 10^6^ CFU/g in the case of PY0001 *ena1-Δ0/ena1-Δ0*) in the fecal pellets. The CFU/g values varied considerably, but only in the case of a single mouse and a single timepoint was the CFU/g value below detection. Mean CFU/g values for the two yeasts were ~8.49 × 10^4^ (S.D. ~7.10 × 10^4^) and 2.10 × 10^5^ (S.D. ~2.62 × 10^5^) on days 1–14 for PY0001 and PY0001 *ena1-Δ0/ena1-Δ0*. There was no statistical difference between the two yeasts neither during the gavage experiment (days 1–14), nor after the gavaging (days 15–18) when the yeast CFU number rapidly declined in the case of both yeast samples, in most cases to below detection level (Fig. 8).

### Avirulent *S. ‘boulardii’* strain (PY0001 *ena1-Δ0/ena1-Δ0*) showed comparable effects on gut microbiome as the original probiotic yeast

**Fig. 9:**
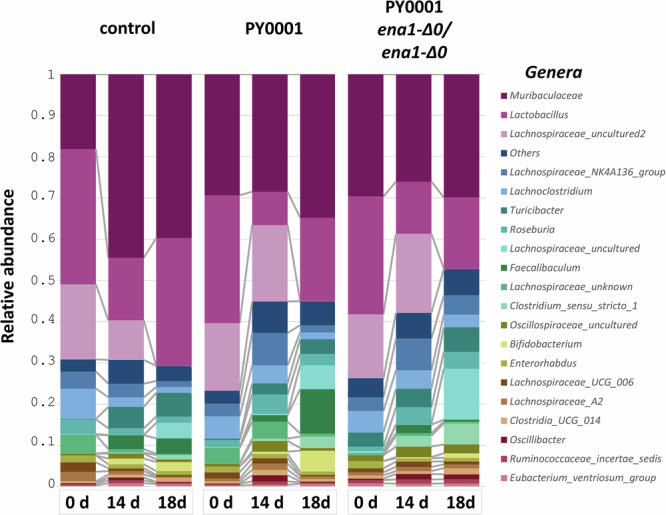
The effect on probiotic yeast gavaging on the mouse gut bacterial microbiome. Genus-level bacterial taxa (color-coded) and their relative abundance in the control and the two yeast-treated mouse groups before gavaging (0 d), a day after the stop of daily gavaging (14 d), and five days after the last gavaging (18 d). Data on bacterial abundance means from all animals is shown. The top 20 most abundant taxa are displayed, and less abundant taxa are merged as “Others”. Number of mice fecal samples used for the analysis was *n* = 8, one from each mouse, in each of the three groups at each time-point.

During the gavaging experiments, fecal samples were also used to isolate DNA and to identify bacterial microbiome through 16S metabarcoding. Fecal samples from all control and yeast-gavaged mice were subjected to this analysis. Samples were collected on day 0 just before the first gavaging, the day after last gavaging (14 d), and five days after last gavaging (18 d). The most abundant genus-level taxa were an unidentified Muribaculaceae, *Lactobacillus*, and an unidentified Lachnospiracae taxon (Fig. 9, Supplementary Fig. 9). Changes in bacterial microbiota were apparent among the samples. Figure 9 shows relative abundance data averaged across mice in the control and two yeast-treated groups. Lactobacilli in all three groups decreased in abundance from day 0 to day 14, and increased from day 14 to 18, while other taxa showed a variable picture. Analyses of alpha (Supplementary Table 15) diversity showed that the mouse groups were similar at the start of the experiment in the case of the control group and the PY0001-treated group, and the PY0001 *ena1-Δ0/ena1-Δ0* treated mice were more diverse than the control group. Groups at the end of gavaging did not differ. Start and end of gavaging samples in each group differed significantly (Supplementary Table 15), as well as end of gavaging and end of experiment samples in each group—alpha diversity increased when the yeast was gavaged, and decreased when gavaging stopped in all groups. Regarding beta diversity, the mouse groups did not differ at the start of the experiment (Supplementary Table 16). After 14 days, both yeast-treated mouse groups’ bacterial microbiota differed from that of the control group, but not from each other. Beta diversity at the start and end of gavaging differed significantly in all groups, as did end of gavaging vs. end of experiment in each group.

## Discussion

Reports about infections of probiotic origin are on the rise in the recent years and probiotic yeast fungemia cases are no exception^6,8,9,36,37^. Several *S. ‘boulardii’* clinical isolates show virulence in fungemia mouse model demonstrated by high lethality and kidney burden, yet to our best knowledge, only a few have been done to date to reveal pathogenicity determinants of this probiotic yeast. Thus, our goal was to identify some of the genetic traits that are responsible for the yeast becoming pathogenic (specifically in fungemia cases) through applying a mammalian model and stress phenotyping.

First, immunosuppressed BALB/c mice were infected and followed for 6 days with 14 different *S. ‘boulardii* yeast isolates, originating from commercial and clinical sources. The commercial isolates were avirulent (87%–100% mouse survival), however some clinical isolates (465/2018, 2251/2018) had remarkable virulence shown by 28% and 42% survival of the applied mouse model, respectively (Fig. 1). It is important to note that clinical isolates were derived from different niches of the human body (Table 1), hence their potential genomic and phenotypic adaptations, triggered by the human host, might differ from one another^27^, resulting in varying level of virulence in an animal model. This can be the reason why bloodstream infections of our mouse model with some clinical isolates result in low lethality, while the 465/2018 and 2251/2018 isolates show high lethality in mice (Fig. 1). Additionally, higher lethality usually occurred in the case of isolates showing high kidney burden. In case of the avirulent isolates (PY0001, PY0002, DE27020, DE35762) the median kidney burden was in the range of ~10^2^–10^3^ CFU/g, while isolates that caused high lethality (465/2018, 2251/2018, DE45866, DE42533) showed remarkably high median kidney burden in the range of 2–2.7 × 10^5^ CFU/g (Fig. 2). These results suggest that the level of kidney burden is a valid proxy to assess the virulence of yeast strains and isolates, at least when a shorter immunosuppression is applied.

To uncover the phenotypic adaptations that enabled some isolates to be virulent, we decided to test the stress tolerance of two commercial isolates (PY0001, PY0002), two isolates that showed high virulence in our mouse model (465/2018, 2251/2018), and the two blood isolates (DE6507, DE35762). Our approach involved first the isolation of in vivo and in vitro subclone lineages of the commercial and clinical isolates, and this large number of subclones was used to assess what types of stress factors are most likely being under strong selection in the mammalian host. Plates were supplemented with amphotericin B, fluconazole, Congo Red, NaCl, and LiCl in spot-plate stress tolerance assays. This experiment revealed that subclones of isolate 465/2018, that caused the highest lethality among the mice, were highly sensitive to Congo red, and had marked NaCl tolerance both before and after mouse infection (Fig. 3b). Furthermore, all tested clinical isolates showed elevated LiCl tolerance, which increased even more (more subclones had tolerance score of 3 or 4 compared to the YPD subclones) after mouse infection and re-isolation (Fig. 3b). This shows that those isolates that were already exposed to the stress conditions present in the human host tended to adapt towards higher LiCl tolerance. Based on these results, we suspected that the high level of virulence might be correlated to genes and pathways also important in Li^+^ and Na^+^ ion tolerance. These ions are exported by the Nha1 ion antiporter or the Ena1 ion pump in yeast, respectively, and are involved in tolerance of various stresses, including alkaline pH^32–34^. Thus, in the next phase of our study, we targeted their respective genes, *NHA1* and *ENA1* in the probiotic yeast, utilizing our isolate collection to represent different *S. ‘boulardii’* genetic backgrounds that differed mostly in heterozygosity but not in structural variations, to study the effect of gene deletion. Using comparative genomics, we established that these genetic editions generally did not result in unintended consequences in the genomes of the isolates. Merely one large segmental duplication was found in one strain (right arm of chr. XVI), and a ~ 30 kb sized deletion was uncovered in three modified strains (two *NHA1* and one *ENA1* mutant). Heterozygosity and other features of the strains remained remarkably consistent after the CRISPR/Cas9-based deletion of the two target genes.

The *NHA1* and *ENA1* deletion mutants obtained in this study were injected through the lateral tail vein into chemically immunosuppressed BALB/c mice to simulate fungemia, then lethality and kidney burden were monitored and compared with the results that were gained in the case of the wild type isolates in experimental infection lasting 6 days (Fig. 4a, b). Based on our results, the deletion of *NHA1* resulted in a surprising increased virulence in the case of the three genetic backgrounds, while it decreased in the case of two (Fig. 4b). Notably, median kidney burden followed the same pattern in the case of these yeasts (Fig. 4b). Thus, the *NHA1* deletion did not unequivocally result in potentially safer probiotic strains. These results also highlight the usefulness of deleting a gene not just in one but multiple, yet closely related, genetic backgrounds. Epistasis and other phenomena may have a considerable effect on how phenotype and virulence is affected by the perturbations of the yeast genome during genetic modifications, similarly to our recent observations on the effect of *HMX1* deletion in six *S. ‘boulardii’* genetic backgrounds^30^.

Mouse survival in the case of the *ENA1* deletion mutants, however, was invariable across the six genetic backgrounds and amounted to 100% across all strains during the 6-day long infection experiments, and median kidney burden values were under 2 × 10^4^ CFU/g, which is significantly lower than the values observed in the case of the highly virulent 465/2018 and 2251/2018 wild type isolates (Fig. 4a, b). Additionally, *ENA1* deletion resulted in significant growth defect in the presence of LiCl, and NaCl stress. Thus, *ENA1* contributed not only to Li^+^ and Na^+^ tolerance but also to the virulence of our probiotic yeast isolates. This means that these engineered strains have a highly reduced potential to become pathogenic in various *S. ‘boulardii’* genetic backgrounds and hence, they may offer a safer way of application to patients as probiotic supplements. The study and plausibly, the commercialization of similar designer probiotics has gained tract recently^11,12,17^, hence, we also tested and showed that the modified knockout strain of PY0001 doesn’t show diminished growth capability in/on standard yeast media and at pH values up to pH 7.5. The antimicrobial activity of the knockout strain also matched that of the parent isolate, and in the in vitro tests, stemmed from acidification of the culture supernatants. Furthermore, according to our results, *ENA1* can also be deleted from the probiotic yeast genome in a way that enables the heterologous expression of therapeutic molecules, as shown with the antilisterial peptide Leucocin C in this work. Although biotherapeutic strains with heterologous protein or peptide production still need further research for efficiency and applicability, our current results showed that the *ENA1* deficient *S. ‘boulardii’* yeast is promising for such applications, as it can produce a heterologous peptide in vitro and its growth in vitro is not diminished (Fig. 7, Supplementary Figs. 4–5 and Supplementary Tables 7–11). Regarding safety in human therapeutic applications, the antifungal drug MIC values were variable among the PY0001 probiotic isolate and its modified strains, but remained comparably low when the range of tested concentrations in the CLSI protocol is considered (Supplementary Table 12). The 21-day-long survival tests with the wild-type probiotic yeasts and the knockout and LecC-substituted strains further show that strain engineering described in this work produced yeasts with dramatically reduced chance to cause fungemia (Supplementary Fig. 3) even during prolonged immunosuppression in mice. If the results are found to be translatable for human use in future studies, these yeasts are considered potential probiotics and/or biotherapeutics that survive in the host GI tract and similarly affect the host’s gut microbiome. Similar designer probiotics could unify safety and biotherapeutic opportunities in future applications.

These results show that the development of avirulent strains was achievable by first studying in vivo selection of stress resistance phenotypes and the subsequent deletion of the *ENA1* gene, but not unequivocally by the deletion of another stress gene, namely *NHA1,* in the case of the probiotic yeast. *ENA1* knock-out strains have the potential to be used as a probiotic with reduced risk of causing life-threatening infections, especially in patients susceptible to fungal infections. Such strains can also be utilized as platform strains for genetic engineering goals that have already been achieved with other strains of unknown and unassessed pathogenic potential, as well as for future genetic engineering aspirations. Future research should be aimed to test novel, therapeutic molecule-producing versions of the safe probiotic yeast in vivo against gastrointestinal pathogens and in animal disease models. We argue that a combination of safety and therapeutic usefulness is the future direction of designer probiotics.

## Materials and methods

### Yeast isolates and patient data in this study

In this study, four commercial isolates of the *S. ‘boulardii’* were used, originating from two batches each of two different probiotic supplements. For the ten clinical isolates used, detailed patient and isolation data were available^5,27,31^, and they were collected from the university clinics of Debrecen and Szeged in Hungary (Table 1). Patient data were handled in accordance with EU, state, and local regulations, with a clinical study ethics approval from the Regional and Institutional Research Ethics Council of Debrecen (DE RKEB/IKEB 5194-2019). Gene deletions were performed using CRISPR/Cas9 genome editing in the case of two commercial (PY0001, PY0002) and four clinical (DE6507, DE35762, 465/2018, 2251/2018) yeast isolates (Table 1.). Stocks of the commercial and clinical isolates and deletion mutants were saved at −70 °C in YPD broth (VWR Chemicals, Solon, OH, USA, pH 5.8) supplemented with 30% v/v glycerol. Subculturing was minimized to prevent the geno- and phenotypic changes in the original isolates^38^ except for the isolation of in vitro subclone lineages. In the latter case, the commercial and clinical isolates were all cultured in 5 mL YPD liquid medium for 24 h at 30 °C with 180 rpm shaking in culture tubes, and a dilution series was plated onto YPD agar plates. Individual colonies were randomly selected and given identifiers referring to original isolate and subclone lineage, these were also saved to our culture collection.

### Lethality and kidney burden in immunosuppressed BALB/c mice and the isolation of in vivo subclones

For the kidney burden experiments, BALB/c immunocompromised female mice (*n* = 7–9 per isolate, *n* = 112 in total; 21–23 g body weight; Charles River Laboratories, Veszprém, Hungary) were used. In case of PY0001, PY0002, 465/2018, 2251/2018, DE6507, DE35762 kidney burden data were used, published by Imre et al.^30^. In the cited article, this sample size and experimental setup were found to enable comparisons between *S. ‘boulardii’* isolates. All results from all experimental animals were used; none were excluded. The animals were maintained in accordance with the Guidelines for the Care and Use of Laboratory Animals. The experiments were approved by the Animal Care Committee of the University of Debrecen, Debrecen, Hungary (permission no. 12/2014 DEMÁB) and were done in accordance to the European Union guidelines (2010/63/EU) in Debrecen. Mice were housed 4 per cage, were provided with standard chow (SM Rat/Mouse, Breeding and Maintenance, 10 mm, autoclavable) and water *ad libitum*, and maintained on an artificial cycle of 12-h light and 12-h dark. Mice were acclimatized for one week before the experiment. Immuno-suppression was achieved by intraperitoneal administration of 150 mg/kg cyclophosphamide 4 days prior to infection, 100 mg/kg cyclophosphamide 1 day prior to infection, 100 mg/kg cyclophosphamide 2 days post-infection, and 100 mg/kg cyclophosphamide 5 days post-infection. Animals were infected intravenously through the lateral tail vein with a single puncture. The inoculum contained 1–1.5 × 10^7^ yeast cells in 0.2 mL physiological saline and for each isolate and mutant strain 7–9 mice were challenged. Inoculum density was confirmed by plating serial dilutions on Sabouraud dextrose agar. Survival of the mice were followed for 6 days post-infection, then surviving mice were euthanized, and kidneys were removed and homogenized aseptically. In case of severe signs of kidney failure and previously established endpoint criteria (permission no. 12/2014 DEMÁB, monitored daily), mice were euthanized by CO_2_ and cervical dislocation. Kidney burden was determined by serial dilutions of the homogenized kidneys in physiological saline on YPD agar plates. Colonies were counted after 2 days of incubation at 37 °C to determine the number of living cells in the kidneys. The individual colonies were saved into our culture collection as described above and were given identifiers referring to inoculated wild-type isolate, mouse specimen, and individual colony. Thereby we possessed the original probiotic, clinical isolates, the subclone lineages from YPD medium cultures, and in-host evolved subclone lineages as well. Until the 6^th^ day post-infection, survival of the inoculated mice was followed, and the data was used to generate 6-day survival curves which were compared as described in the Statistics section. Furthermore, 21-day survival data was recorded for six isolates and their respective *ena1-Δ0*/*ena1-Δ0* and *ena1::LecC/ena1::LecC* modified strains. Based on results of shorter infection experiments, *nha1-Δ0/nha1-Δ0* mutants were excluded. BALB/c female mice (21–23 g; Charles River) were treated with 150 mg/kg cyclophosphamide 4 days and 1 day prior to infection, and with 100 mg/kg cyclophosphamide every 3 days until the 21^st^ day post-infection. The inoculum contained 1–1.5 × 10^7^ yeast cells in 0.2 mL physiological saline and for each isolate 8 mice were used. Inoculation was performed as in the kidney burden experiment. Survival was recorded daily, and data was used for Kaplan-Meier analysis as described below. Since only survival and CFU values were recorded in these experiments, blinding was not used, inter-cage effects were not tested due to relatively low sample size.

### Spot plate stress phenotyping

The presence of slightly different genetic lineages within a strain, clonal heterogeneity, and selection inside the mouse host acting upon these was taken into our focus in phenotyping. Ten in vitro subclones of wild type isolates were tested as controls, along with 19–20 in vivo selected subclones. Prior to spot-plate stress phenotyping yeasts were pre-cultured on YPD agar plates for 24 h. For the experiments, 0.5 MacFarland cell suspensions were prepared, and four 10 µl drops per isolate/subclone were inoculated in three replicates. The drops contained ~10^4^, 10^3^, 10^2^, and 10 cells. Stress conditions, based on Strope et al.^39^, were tested on synthetic defined (SD) agar plates (6.7 g/L yeast nitrogen base without amino acids, 2% glucose, 2% agar), supplemented with one of the following stressors: 0.125 µg/mL amphotericin B, 15.00 µg/mL fluconazole, 16.00 µg/mL Congo red, 342.23 mM (2 w/v %) NaCl, or 50 mM LiCl. Plates were incubated at 37 °C for 3 days, then photographed to document the number of droplets showing growth. Tolerance scores were determined based on spots showing growth as follows. Score 0: no visible growth; 1: growth of the first spot (containing 10^4^ cells); 2: growth of two spots (containing 10^4^ and 10^3^ cells); 3: growth of three spots (containing 10^4^, 10^3^, and 10^2^ cells); 4: growth of all 4 spots (containing 10^4^, 10^3^, 10^2^, and 10 cells).

### Gene deletion constructs, Leucocin C gene integration, and mutant selection

Benchling (https://www.benchling.com/) was used to design HDR templates and gRNA oligonucleotides for the gene knock-out experiments, considering the work of Akhmetov et al.^40^. Repair DNA was prepared by PCR reaction using Dream Taq Green polymerase (Thermo Fisher Scientific, Waltham, MA, USA). Each repair DNA included the same 20 bp insertion cassette and had uniquely designed homologous sequences to knock out the genes *ENA1* and *NHA1*. Both of these are single-copy genes in *S. ‘boulardii’*^30^. Oligonucleotide sequences of repair DNA and gRNA are presented in Supplementary Table 17. The assembly of the plasmid coding for the Cas9 nuclease and gRNA was carried out using the MoClo Yeast Toolkit from Addgene (YTK, Addgene kit, Addgene, Watertown, MA, USA; catalog number: 1000000061)40^40,41^. DH5α chemically ultra-competent *Escherichia coli* cells were used for all cloning steps. Cells were selected after transformation on lysogeny broth (LB, Miller) agar plates containing the appropriate antibiotics (0.1 mg/mL ampicillin, 0.025 mg/mL chloramphenicol, or 0.05 mg/mL kanamycin). Ultra-competent *E. coli* cells were prepared with the Inoue method^42^ and transformations were carried out with the heat shock method^43^. Plasmid isolations were carried out with the GeneJet Plasmid Miniprep Kit (Thermo Fischer Scientific). Transformation of the HDR templates (1000 ng) and the Cas9/gRNA coding plasmid (200 ng) into yeast were performed according to Lee et al. with modifications^30,39^. Selection of the transformed yeasts was done by using YPD plates (VWR Chemicals) supplemented with 0.1 mg/mL nourseothricin. Then multiple colonies were inoculated onto YPD agar medium to promote the loss of the Cas9 and gRNA coding plasmid carrying the nourseothricin acetyltransferase (NAT) antimycotic resistance gene. Verification of successful gene deletions were done by colony PCR, with GoTaq G2 Hot Start polymerase (Promega, Madison, WI, USA). Both the lack of the targeted gene’s ORF and the correct insertion of the HDR templates were checked. The oligonucleotides used for verification are listed in Supplementary Table 17. For gel electrophoresis, 1% low electroendosmosis (LE) agarose gel (Promega, Madison, WI, USA) was used, with Tris Acetate EDTA (TAE) buffer.

A version of the *ENA1* knockout mutant with the integration of the Leucocin C (LecC) gene at the position of the gene was also designed. Leucocin C is a class IIa bacteriocin produced by a lactic acid bacterium effectively inhibiting the growth of *Listeria monocytogenes* and a synthetic version of this peptide’s gene has already been heterologously expressed in the probiotic yeasts from an expression plasmid (thus without chromosomal integration)^12^. In this study, a plasmid carrying the DNA fragment for the leucocin C secretion was synthesized by Integrated DNA Technologies (Coralwille, IO, USA). The sequence included the *S. cerevisiae* α-mating factor signal sequence to guide the extracellular secretion of the LecC peptide (Supplementary Table 17)^12,44^. A set of PCR primers (Supplementary Table 17) were used to add the above-described homologous sequences upstream and downstream to the synthetic fragment enabling integration by homologous recombination to the *ENA1* locus. The *ENA1* gene was then targeted with the CRISPR/Cas9 method using the same guides as described above. For transformation 1000 ng LecC synthetic fragment and 200 ng CRISPR plasmid was used. Primers for homologous regions added to the LecC cassette and for verification of integration are also described in Supplementary Table 17 along with whole-genome sequencing based analyses on the *ena1::LecC/ena1::LecC* strain (Supplementary Tables 2-4).

### Whole genome sequencing and analysis

To compare the genomes of the modified strains and the original isolates, and to search for potential off-target effects and genome structure variations of the knockouts, we applied short-read sequencing and comparative genomics. Genomic DNA was isolated after 24 h growth of freshly revived stocks streaked onto YPD and incubated at 30 °C. Library preparation was performed using tagmentation with the Nextera DNA Flex Library Prep kit (Illumina, San Diego, CA, USA) according to the manufacturer’s protocol. Sequencing was performed using 150 bp paired-end reads on an Illumina NextSeq 500 system, with approximately 50–60× coverage of the nuclear genome. Raw reads were deposited to NCBI SRA under BioProject PRJNA1165191. The original isolates have been sequenced before, and those reads were used here^30^. Details of the genomics pipeline correspond to our previous analysis^30^ and are described in Supplementary Table 18 in detail. Briefly, filtered and trimmed reads were mapped to the PY0001 reference genome (ASM2473226v1)^30^, alleles were called to determine heterozygous positions, then they were compared to highlight differences among wild type isolates and modified strains. Allele ratios were plotted along the chromosomes. Coverage mapping with 10 kb windows sliding every 5 kb was used to identify large structural variations and for visualizations. Per-base coverage comparison was used to locate the exact loci of large deletions and segmental duplications and to visualize the CRISPR/Cas9 deleted loci’s neighboring regions. A draft assembly was also produced for PY0001 *ena1::LecC*/*ena1::LecC* to confirm correct integration of the leucocin C gene to the targeted *ENA1* locus (Supplementary Table 18). Sequencing reads’ metadata is shown in Supplementary Table 19.

### Tolerance of gastrointestinal conditions, high pH, bile salts, and antifungals of modified strains

To conduct an assessment on whether gene deletion and the integration of LecC diminished the potential probiotic applicability of the most promising modified strains, three samples were subjected to several growth and survival tests. The yeasts PY0001, PY0001 *ena1-Δ0/ena1-Δ0*, and PY0001 *ena1::LecC*/*ena1::LecC* were inoculated onto YPD agar buffered to various pH values with physiological relevance in the gastrointestinal system. The above-described spot plate assay was applied, growth was recorded after 2 days of incubation at 37 °C. The used pH values in YPD plates were^45^: pH 6.1 (as in duodenum, using MES 50 mM buffer); pH 7.1 (as in middle small intestine, using Tris 50 mM buffer), pH 7.5 (as in distal small intestine, using Tris 50 mM buffer), and pH 6 (as in cecum, using MES 50 mM buffer), along with a pH of 8.0 (using Tris 50 mM buffer) representing pronounced alkali stress for yeasts. Buffered media were filter-sterilized and mixed with an autoclaved agar solution (cooled to 65 °C) to reach desired end concentrations of buffers and YPD components. Bile salts (bile salts suitable for microbiology, Sigma Aldrich) were also added to SD agar medium (cooled to 65 °C) from a filter-sterilized stock solution to reach concentrations of 0.1, 0.05, and 0.025 w/v %, and spot plate inoculations were carried out as described above. Additionally, yeasts were subjected to the INFOGEST 2.0^46^ in vitro simulation of gastrointestinal digestion protocol. Simulated salivary, gastric, and intestinal fluids with digestive enzymes and bile were prepared as described in Brodkorb et al.^46^. A yeast suspension of 5 MacFarland units was prepared in water, and a dilution series was plated onto plate count agars (PCA, from VWR) to calculate original cell density. From the undiluted suspension, 25 µl was mixed in wells of a 96-well plate, and the suspension was mixed with 25 µl of simulated salivary fluid. After 2 min of incubation at 37 °C, 50 µl of simulated gastric fluid was mixed to the samples and incubated for 2 h at 37 °C, followed by the addition of 200 µl of simulated intestinal fluid and incubation for 2 h at 37 °C. Samples were taken thereafter, diluted with physiological saline, and plated onto PCA plates to count colonies after 3 days of incubation at 37 °C. The percentage of surviving colony forming units after the INFOGEST 2.0 simulated digestion was calculated; each yeast was subjected to the test in triplicate.

Furthermore, in order to assess whether any of the mentioned modified strains showed undesirable decreased antifungal susceptibility, we assessed susceptibility to fluconazole, amphotericin B, anidulafungin, caspofungin, and micafungin (all from Merck, Budapest, Hungary) in YPD (2% glucose, 2% peptone, 1% yeast extract) medium using the broth microdilution method, following the Clinical and Laboratory Standards Institute (CLSI) M27-A3 guideline^47^. According to the guideline, the microdilution assay should be performed in RPMI-1640 medium; however, *S. ‘boulardii’* yeasts did not exhibit growth in this medium. Partial inhibition (≥50% growth reduction vs. growth control) was used for fluconazole and echinocandins, while complete inhibition (100% growth reduction) was applied for amphotericin B.

### Analyzing duplication time, biomass production, growth on standard yeast media, and liophilization tolerance for the modified strains

To conduct a preliminary assessment on whether gene deletion and the integration of LecC diminished the usability of the most promising modified strains as potential probiotic products, tests of growth and liophilization were carried out. The yeast PY0001 and its modified strains PY0001 *ena1-Δ0/ena1-Δ0* and PY0001 *ena1::LecC*/*ena1::LecC* were inoculated into small-scale liquid cultures in 250 mL Erlenmeyer flasks with 100 mL YPD medium, started with OD_600_ = 0.1 (optical density) of cells, in triplicates. The flasks were shaken at 180 rpm at 37 °C for 24 h with regular OD measurements, after which cells were collected with centrifugation (6000×g) and dried on filter paper (55 °C, 8 h). Total dry cell mass was measured. From the OD measurements, duplication time in the exponential phase was calculated. Samples were also inoculated onto YPD agar (non-buffered), SD agar, and malt-extract (ME) agar using the above-described spot plate assay, growth was recorded after 2 days of incubation at 37 °C. Samples from the same liquid culturing setup as above were also subjected to liophilization in a suspension of 120 g/L milk powder and 70 g/L trehalose. The suspension was frozen at –70 °C for 2 h, then lyophilized for 3 h in triplicates^48^. Subsequently, viable cell number was checked by plating dilution series to PCA along with a dilution series of the original suspension to calculate the ration of surviving cells.

### Antagonism tests against bacteria

As earlier studies have shown antimicrobial activity of the probiotic yeast^14^, we tested whether this activity was affected by gene deletion using bacterial strains listed below: *Bacillus subtilis* ATCC6051, *Escherichia coli* ATCC11775, *Klebsiella* sp. UDeb-VGB2, *Listeria monocytogenes* NCAIM B.01934, *Pseudomonas putida* group UDeb-VGB1 (strains UDeb-VGB1 and 2 are unpublished and originate from our strain collection of foodstuff-derived bacteria). Agar-well diffusion assays were used, plates with 20 mL Nutrient Broth agar medium (HiMedia, Modautal, Germany) were inoculated with sterilized cotton swabs dipped into a 0.5 MacFarland suspension of bacteria in sterile water. After the inoculum dried, holes were punched into the agar using an 8-mm sterile pipette tip, and the bottom of holes were sealed with a droplet of molten medium. Each well was then filled with ∼150 µL of yeast culture supernatant (48 h cultures at 37 °C in triplicates, as described above for biomass production tests). All agar-well plates were incubated at 37 °C for 18 h and then photographed, inhibition zones were measured. For the *L. monocytogenes* inhibition tests, the supernatants were produced the same way, but they were also subjected to concentration of the produced LecC peptides. 40 mL supernatant was precipitated using saturation with ammonium sulfate (40% w/v), with agitation at 20 °C for 30 min. Precipitates were centrifuged at 13000×g for 30 min at 4 °C, then dissolved in 1 mL of 200 mM sodium phosphate buffer (pH 6) according to Li et al. and references therein^12^. Samples were subjected to dialysis in 1 L of 20 mM PBS buffer (pH 7.2) for 24 h using SnakeSkin (Sigma Aldrich) dialysis membranes with a cutoff of 5 kDa. An aliquot of dialysed samples was subjected to peptide degradation as a control using 30 min of incubation at 55 °C with 5 units of Proteinase K (Zymo Research, Irvine, CA, USA). *L. monocytogenes* cultures were inoculated onto Mueller-Hinton agar plates with 5% horse blood (Clinichem, Budapest, Hungary) with cotton swabs as above, and the same agar well diffusion method was used with the dialysed samples as with simple supernatants. YPD controls were used for the diffusion assays. Antagonism tests were carried out with the yeasts PY0001, PY0001 *ena1-Δ0/ena1-Δ0*, and PY0001 *ena1::LecC/ena1::LecC*. As Offei et al.^13^ showed in an earlier study that the in vitro antibacterial effect of the probiotic yeast is predominantly due to the acidification of the medium, we also tested supernatants after their pH was set to 6.0. As Offei et al.^13^ showed in an earlier study that the in vitro antibacterial effect of the probiotic yeast is predominantly due to the acidification of the medium, we also tested supernatants after their pH was set to 6.0 using 1 M NaOH.

### Mouse gavaging with yeasts

The gavaging experiment with the yeasts PY0001, PY0001 *ena1-Δ0/ena1-Δ0*, and PY0001 *ena1::LecC/ena1::LecC* was approved by the NC State University Institutional Animal Care and Use Committee (IACUC; protocol ID: 23-434). To test viability and clearance in the gut, 8-week-old female C57BL/6 J mice (*n* = 24 total, *n* = 8 per group, mice randomly assigned to groups upon arrival) with normal microbiome (i.e., neither germ-free, nor specific-pathogen-free animals) and immune status were used. Mice were provided with standard chow and water *ad libitum* and maintained on an artificial cycle of 12-h light and 12-h dark. Mice were housed four per cage, and their weight was recorded daily. Mice were acclimatized for one week before the experiment, then gavaged orally every day for 14 days with 10^8^ cells of PY0001 and PY0001 *ena1-Δ0/ena1-Δ0* suspended in 200 µl filtered and sterilized water. Inoculum for one mouse was prepared by setting 5.0 McFarland cell suspension in 10 mL sterile water measured by McFarland densitometer, followed by centrifugation step (4000 rpm, 5 minutes), then suspension of cell pellet in 200 µl filtered and sterilized water. One group received 200 µl water as a control for the duration of the whole experiment. Cell number of gavaged yeast was assessed each day by plating the suspension on yeast peptone dextrose (YPD) agar. To assess CFU number, fecal pellets were collected on day 1, day 5, day 10, and day 14. Between day 15 and 18, pellets were also collected to determine yeast clearance from the gut. Mice were euthanized by CO_2_ and cervical dislocation on day 19. The gavage experiment and feces collection were performed in a biosafety cabinet with sterile instruments, and sterile absorbent pads were used in the gavaging area, in case when pellets were dropped before collection. Weight of the pellets were measured, then pellets were suspended in 1 mL phosphate buffered saline (PBS) before plating for CFU and before DNA isolation for 16S metabarcoding sequencing. To determine CFU, samples were diluted 10x, then 50 µl suspension was plated on Dichloran Rose Bengal Chloramphenicol (DRBC) agar (Thermo Fisher Oxoid) and plates were incubated at 30 °C for 2 days. CFU counts were determined as CFU/g fecal pellet. No data points were excluded and blinding was not applied. Inter-cage comparisons were not made due to the relatively low sample size.

### Bacterial metabarcoding of mouse feces in feeding experiments

For 16S metabarcoding the undiluted fecal samples from days 0 and 14 obtained as described above were centrifuged at 12,100 rcf for 5 minutes and the pellet was used for DNA isolation with QIAamp PowerFecal Pro DNA Kit (Qiagen LLC, USA) according to the manufacturer’s instructions. Isolated genomic DNA samples were sent for 16S (V3/V4 region) metabarcoding analysis to SeqCenter LLC (Pittsburgh, PA, USA) using Illumina short-read sequencing as a paid service with at least 50,000 reads per sample. Data processing, statistics, and visualization are described in Supplementary Table 18. The sequencing files for metabarcoding are deposited under the BioProject number PRJNA1358987.

### Statistics and reproducibility

Statistical analysis was conducted by using the following online tools: VassarStats (http://vassarstats.net/index.html), Astatsa (https://astatsa.com/), and Statistics Kingdom (https://www.statskingdom.com/index.html). In the case of two datasets and normal distribution a two-tailed, two-sample *t*-test (equal variances, Levene’s test) or Welch’s test (unequal variances) was used. In the case of a non-normal distribution, the Mann–Whitney test was used. To compare more than two datasets at once, ANOVA (normal distribution, Levene’s test), followed by Tukey HSD test or Kruskal-Wallis (non-normal distribution), followed by Dunn post-test (corrected for Benjamini-Hochberg FDR) was used to determine which data sets differed significantly. The Log-rank (Mantel-Cox) test was applied for Kaplan–Meier mouse survival analysis. GraphPad Prism 10.2.3. was used for analysis and visualization. Information on sample sizes is indicated in the Supplementary Data File, along with source data underlying the graphs and charts. Experiments were carried out as at least 3 biological replicates, in the case of mouse experiments, at least 7 animals per group were used, sample sizes are noted for each image and graph.

### Ethics permit

The mouse infection experiments were approved by the Animal Care Committee of the University of Debrecen, Debrecen, Hungary (permission no. 12/2014 DEMÁB). Mouse gavaging experiment was approved by the NC State University Institutional Animal Care and Use Committee (IACUC; protocol ID: 23-434). We have complied with all relevant ethical regulations for animal use. Patient data were handled in accordance with EU, state, and local regulations with a clinical study ethics approval from the Regional and Institutional Research Ethics Council of Debrecen (DE RKEB/IKEB 5194-2019). Patient data was solely used to provide context for the origin of the clinical yeast isolates.

### Reporting summary

Further information on research design is available in the Nature Portfolio Reporting Summary linked to this article.

## Supplementary information

Supplementary information

Description of Additional Supplementary Files

Supplementary Data File

Reporting Summary

## Data Availability

Raw sequencing reads used in this study are deposited in NCBI SRA under BioProject PRJNA1165191 and PRJNA1358987. Cohort-called variant files are deposited in FigShare (10.6084/m9.figshare.27105919). Raw data for each graph is included in Supplementary Data File. All additional data are available from the corresponding author on reasonable request.
