# Supplementary material for: ENA1 deficiency attenuates Saccharomyces ‘boulardii’ probiotic yeast virulence in immunosuppressed mouse fungaemia model
Source: Commun Biol. 2026 Mar 6;9:542. doi: 10.1038/s42003-026-09763-z (PMC13096209; doi:10.1038/s42003-026-09763-z)
Supplement: Supplementary file 1 — Supplementary information [file 42003_2026_9763_MOESM1_ESM.pdf]

Supplementary Information

ENA1 deficiency attenuates *Saccharomyces 'boulardii'* probiotic yeast virulence in immunosuppressed mouse fungaemia model

<table>
<tr><th rowspan="3"></th><th colspan="2">Survival</th><th colspan="2">Kidney burden</th></tr>
<tr><th colspan="2">Log-rank (Mantel-Cox) test</th><th colspan="2">Pairwise comparisons (Kruskal-Wallis test)</th></tr>
<tr><th>4 commercial isolates</th><th>10 clinical isolates</th><th>4 commercial isolates</th><th>10 clinical isolates</th></tr>
<tr><td rowspan="4">p-values</td><td rowspan="4">p=0.5283 n.s.
for all isolates combined</td><td rowspan="4">p=0.0026*
for all isolates combined</td><td rowspan="4">p=0.145206 n.s.
for all isolates combined</td><td>p=0.001161 **
for all isolates combined</td></tr>
<tr><td>p=0.003604 **
(DE27020 < 2251/2018)</td></tr>
<tr><td>p=0.002555**
(DE6507 < 2251/2018)</td></tr>
<tr><td>p=0.001234**
(DE35762 < 2251/2018)</td></tr>
</table>

Table S1. Statistical analysis of the differences in virulence in 6 day-long infection experiments of the fourteen wild-type isolates used in this study and in the fungal burden of the kidneys in the mouse infection model. In the case of the Kruskal-Wallis test, Bonferroni correction was applied ($\alpha = 0.005$), and only significant pairwise comparisons are listed. n.s.: non-significant; **: $p < 0.01$. Four commercial isolates: PY0001, PY0002, PY0003, PY0004. Ten clinical isolates: DE27020, DE6507, DE35762, DE3912, DE42807, DE42533, DE45866, 465/2018, 551/2018, 2251/2018. Number of mice used for the experiments was 9 (PY0001, PY0002, PY0004, DE27020, DE6507, DE35762), 8 (PY0003, 551/2018), and 7 (DE3912, DE42807, DE42533, DE45866, 465/2018, 2251/2018).

	I: trisomy	IV: 526,602 – 529,877 homozygous deletion (*ENA1*)	IV: 573,236 – 606,806 heterozygous deletion	V: 488,696 – 565,474 heterozygous deletion	XII: 398,473 – 401,426 homozygous deletion (*NHA1*)	XVI: 547,735 – 931,421 heterozygous segmental duplication
**PY0001**	-	-	-	-	-	-
**PY0001 *nha1-Δ0/nha1-Δ0***	-	-	present	-	present	-
**PY0001 *ena1-Δ0/ena1-Δ0***	-	present	-	-	-	-
**PY0001 *ena1::LecC/ena1::LecC***	present	present, with integration of signal sequence and LecC	-	-	-	-
**PY0002**	-	-	-	-	-	-
**PY0002 *nha1-Δ0/nha1-Δ0***	-	-	-	-	present	-
**PY0002 *ena1-Δ0/ena1-Δ0***	-	present	-	-	-	-
**465/2018**	-	-	-	-	-	-
**465/2018 *nha1-Δ0/nha1-Δ0***	-	-	-	-	present	-
**465/2018 *ena1-Δ0/ena1-Δ0***	-	present	-	-	-	-
**2251/2018**	-	-	-	-	-	-
**2251/2018 *nha1-Δ0/nha1-Δ0***	-	-	-	-	present	-
**2251/2018 *ena1-Δ0/ena1-Δ0***	-	present	-	-	-	-
**DE6507**	-	-	-	-	-	-
**DE6507 *nha1-Δ0/nha1-Δ0***	-	-	present	-	present	-
**DE6507 ΔΔ *ena1-Δ0/ena1-Δ0***	-	present	present	-	-	-
**DE35762**	-	-	-	-	-	-
**DE35762 *nha1-Δ0/nha1-Δ0***	-	-	-	present	present	present
**DE35762 *ena1-Δ0/ena1-Δ0***	-	present	-	-	-	-

Table S2. Structural variants affecting chromosomal regions detected in the genomes of the sequenced wild-type isolates and their respective knockout mutants. Major variants are highlighted in yellow.

	I	II	III	IV	V	VI	VII	VIII	IX	X	XI	XII	XIII	XIV	XV	XVI	Heterozygosity relative to wt
**PY0001**	313	431	226	306	85	115	80	3	98	438	66	171	144	19	99	156	100%
**PY0001 *nha1-Δ0/nha1-Δ0***	294	418	222	305	82	114	74	2	91	408	63	152	139	22	88	150	95.42%
**PY0001 *ena1-Δ0/ena1-Δ0***	306	424	223	292	83	114	71	3	89	432	64	165	135	21	62	149	95.746%
**PY0001 *ena1::LecC/ena1::LecC***	325	440	217	311	95	122	84	12	105	438	68	180	155	31	82	161	102.764%
**PY0002**	288	426	227	300	88	115	79	1	92	430	63	165	136	18	89	151	100%
**PY0002 *nha1-Δ0/nha1-Δ0***	296	385	222	305	83	112	75	3	91	443	63	160	136	15	88	146	98.313%
**PY0002 *ena1-Δ0/ena1-Δ0***	297	417	223	305	83	114	71	0	94	436	65	164	137	23	88	148	99.888%
**465/2018**	302	429	221	313	84	101	77	2	92	434	64	168	141	18	70	65	100%
**465/2018 *nha1-Δ0/nha1-Δ0***	306	428	218	293	81	102	72	2	91	436	62	155	140	17	65	59	97.908%
**465/2018 *ena1-Δ0/ena1-Δ0***	298	433	215	296	85	102	75	1	92	426	62	167	140	19	66	60	98.295%
**2251/2018**	298	420	212	303	84	115	75	3	94	351	65	171	145	26	69	149	100%
**2251/2018 *nha1-Δ0/nha1-Δ0***	307	418	206	299	84	114	71	3	78	347	66	151	142	22	62	146	97.519%
**2251/2018 *ena1-Δ0/ena1-Δ0***	287	421	213	279	85	113	72	2	92	355	66	166	141	23	60	149	97.895%
**DE6507**	291	428	227	308	87	114	76	6	93	439	49	168	144	19	65	43	100%
**DE6507 *nha1-Δ0/nha1-Δ0***	293	427	220	296	85	114	76	16	94	429	47	148	141	16	53	46	97.810%
**DE6507 ΔΔ *ena1-Δ0/ena1-Δ0***	296	427	221	285	80	112	77	5	90	427	48	166	141	19	62	43	97.732%
**DE35762**	308	162	223	309	85	113	77	4	92	440	66	169	146	18	70	154	100%
**DE35762 *nha1-Δ0/nha1-Δ0***	297	160	217	287	80	113	71	4	92	424	65	149	142	16	71	148	95.895%
**DE35762 *ena1-Δ0/ena1-Δ0***	297	159	223	304	84	115	76	3	94	424	66	163	141	16	67	153	97.906%

Table S3. Number of heterozygous variants found in sequenced genomes for each chromosome (indicated with Roman numerals). To aid visual comparisons, stronger shades of blue and red represent lower and higher ends of the observed values, respectively. wt: wild type.

	mtDNA copy number per haploid genome	2µ plasmid copy number per haploid genome
**PY0001**	19.3	15.4
**PY0001 *nha1-Δ0/nha1-Δ0***	22.9	41.7
**PY0001 *ena1-Δ0/ena1-Δ0***	20	43.5
**PY0001 *ena1::LecC/ena1::LecC***	n.d.	n.d.
**PY0002**	16.4	22.9
**PY0002 *nha1-Δ0/nha1-Δ0***	23.1	35
**PY0002 *ena1-Δ0/ena1-Δ0***	21.4	40.9
**465/2018**	24	38.1
**465/2018 *nha1-Δ0/nha1-Δ0***	23.6	40.2
**465/2018 *ena1-Δ0/ena1-Δ0***	20.6	37.5
**2251/2018**	25.3	25.5
**2251/2018 *nha1-Δ0/nha1-Δ0***	22.4	40.4
**2251/2018 *ena1-Δ0/ena1-Δ0***	20.4	38.1
**DE6507**	22.6	25.4
**DE6507 *nha1-Δ0/nha1-Δ0***	19.8	42
**DE6507 ΔΔ *ena1-Δ0/ena1-Δ0***	22.4	38.5
**DE35762**	19.8	27.4
**DE35762 *nha1-Δ0/nha1-Δ0***	26.8	51.8
**DE35762 *ena1-Δ0/ena1-Δ0***	23.1	38.9

Table S4. Copy number variations in mitochondrial genomes and 2µ plasmids per haploid nuclear genome in the wild type isolates and knockout strains. To aid visual comparisons, stronger shades of blue and red represent lower and higher ends of the calculated values, respectively. The PY0001 *ena1::LecC/ena1::LecC* strain was sequenced using a different library preparation kit and resulted in very low mtDNA and plasmid coverage, and was excluded from this analysis.

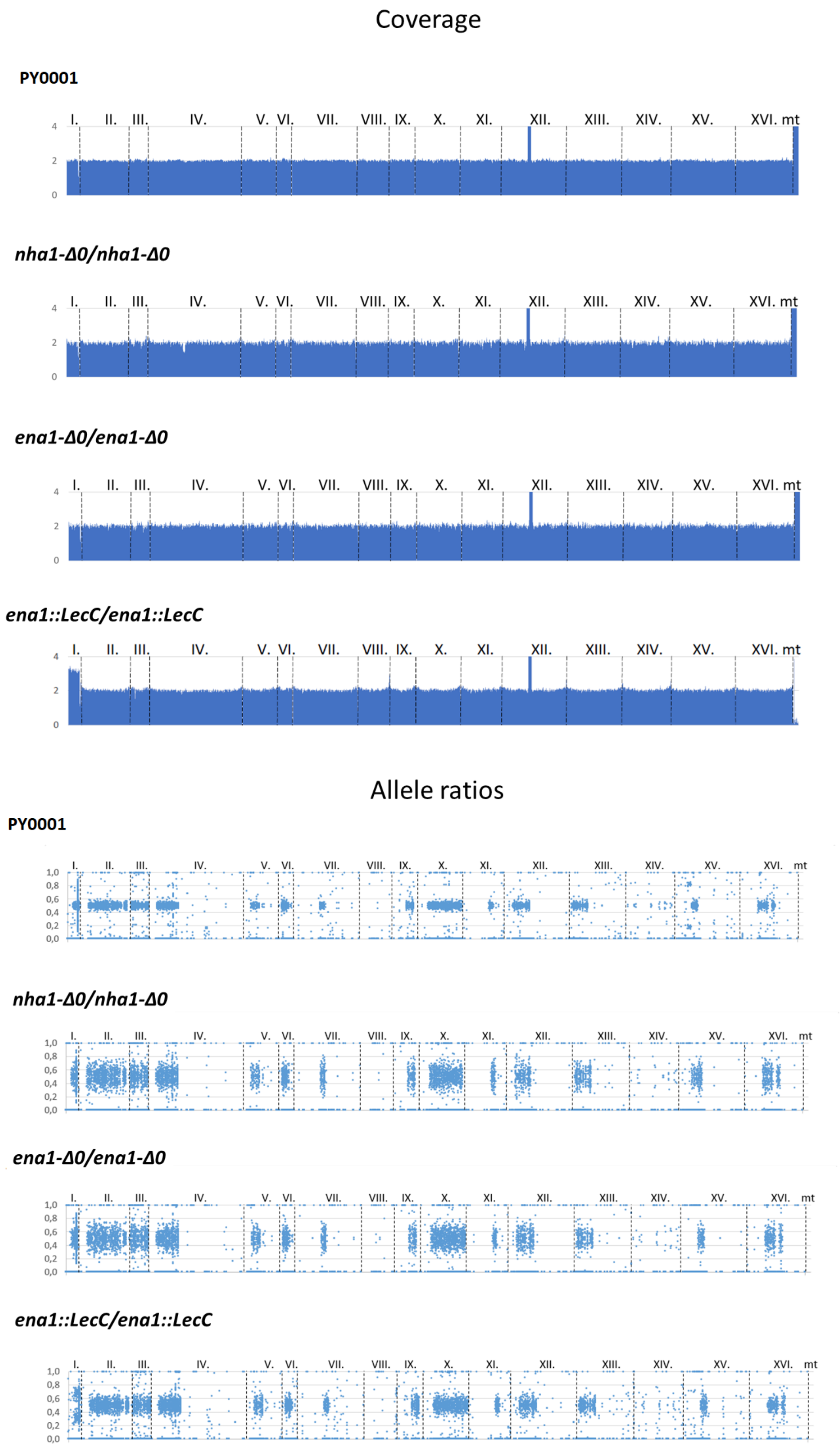

Figure S1a. Coverage and allele plots for PY0001 wild type isolate and the respective knockout strains. Sequencing coverage in 10 kb bins, corrected for ploidy (top parts of the composite images) and allele ratio plots (bottom parts) are all shown along the 16 chromosomes.

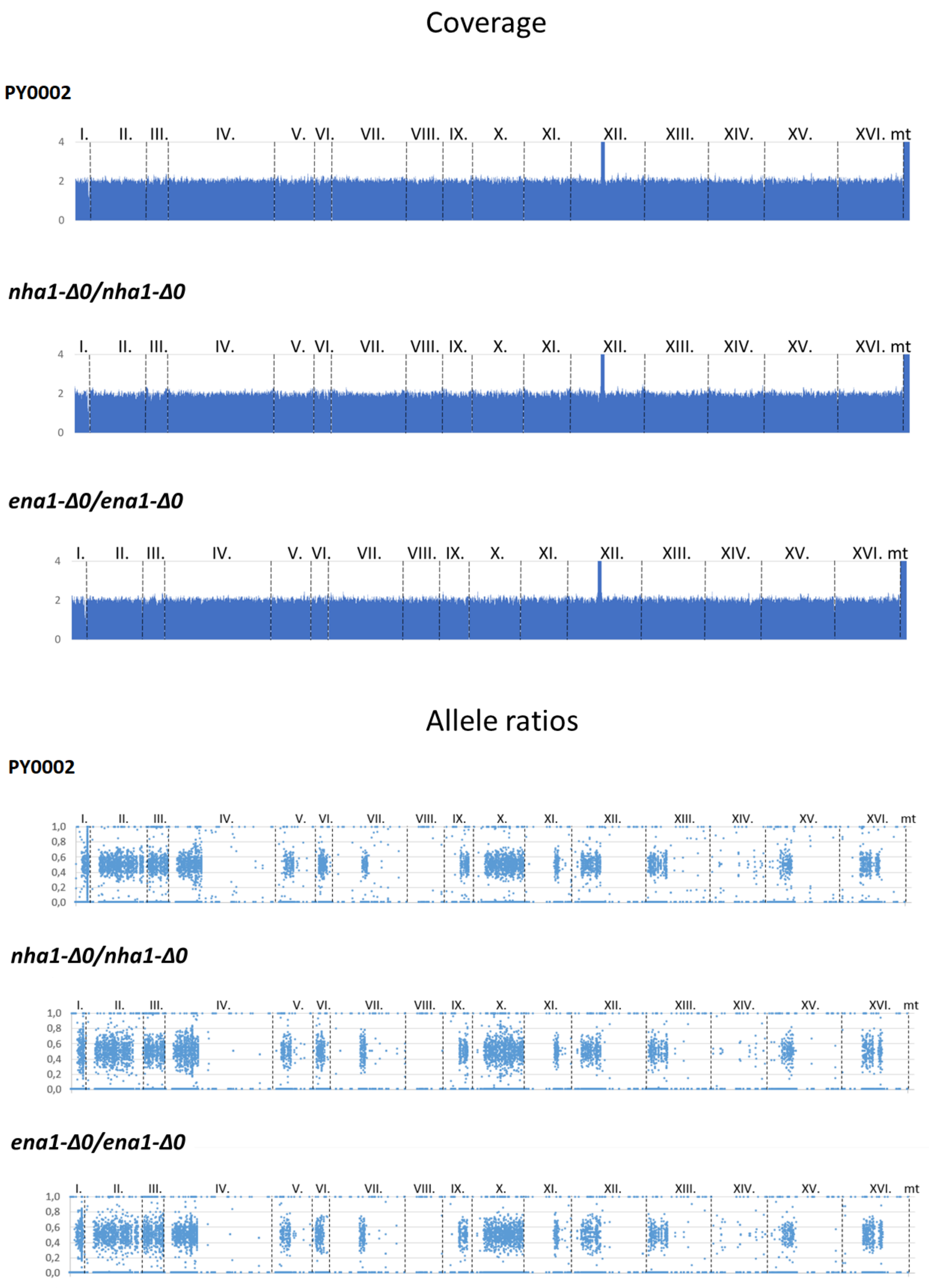

Figure S1b. Coverage and allele plots for PY0002 wild type isolate and the respective knockout strains. Sequencing coverage in 10 kb bins, corrected for ploidy (top parts of the composite images) and allele ratio plots (bottom parts) are all shown along the 16 chromosomes.

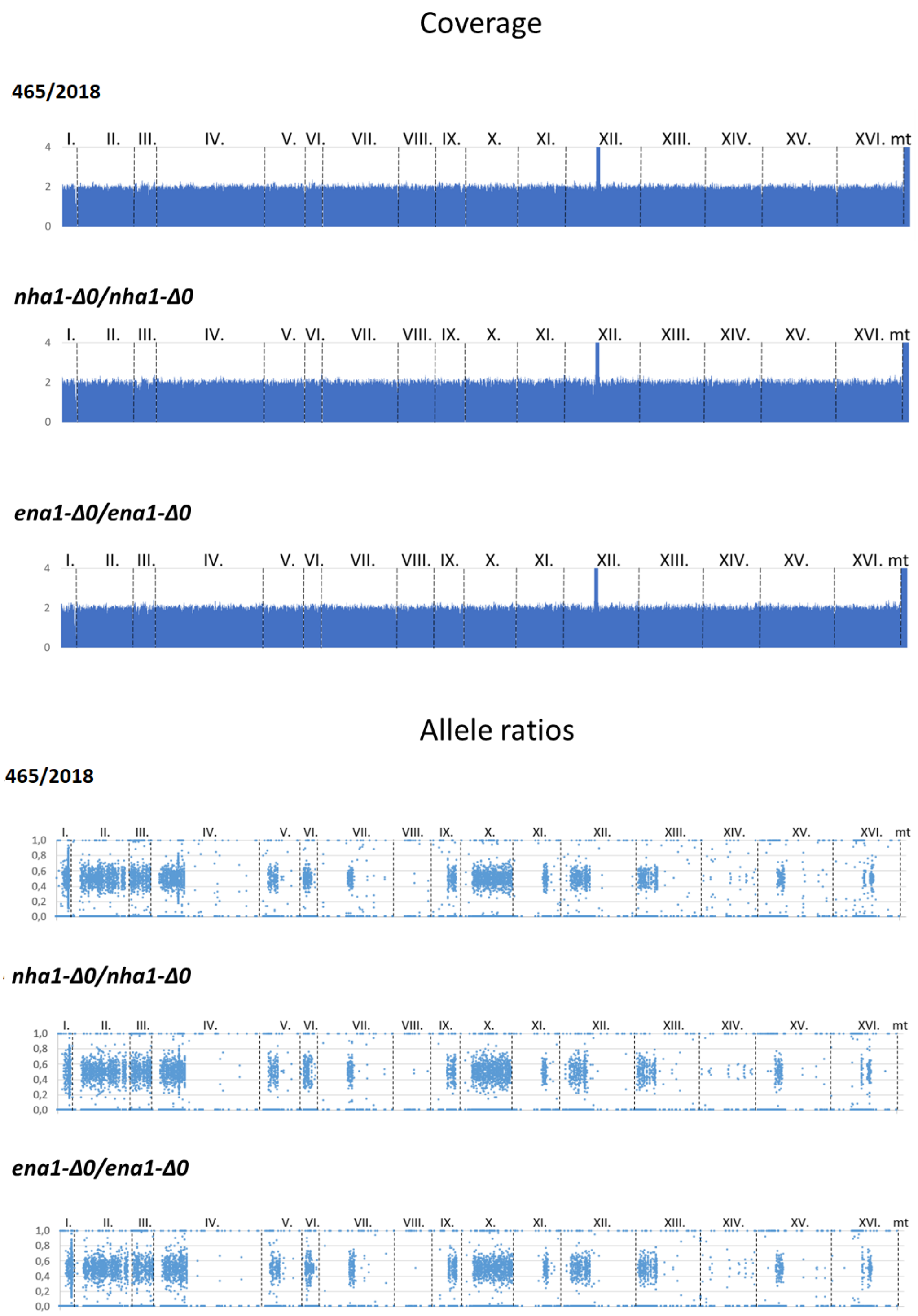

Figure S1c. Coverage and allele plots for 465/2018 wild type isolate and the respective knockout strains. Sequencing coverage in 10 kb bins, corrected for ploidy (top parts of the composite images) and allele ratio plots (bottom parts) are all shown along the 16 chromosomes.

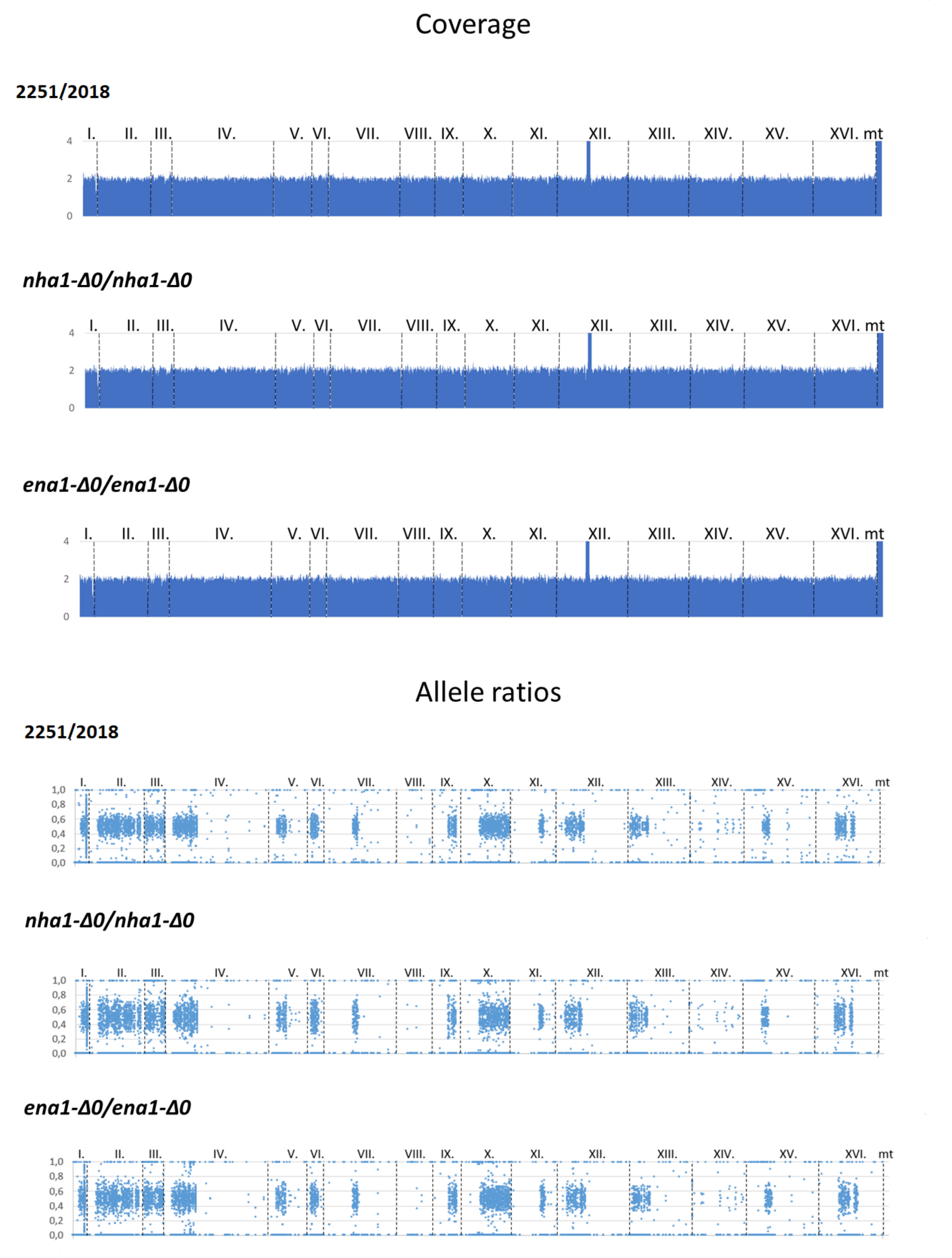

Figure S1d. Coverage and allele plots for 2251/2018 wild type isolate and the respective knockout strains. Sequencing coverage in 10 kb bins, corrected for ploidy (top parts of the composite images) and allele ratio plots (bottom parts) are all shown along the 16 chromosomes.

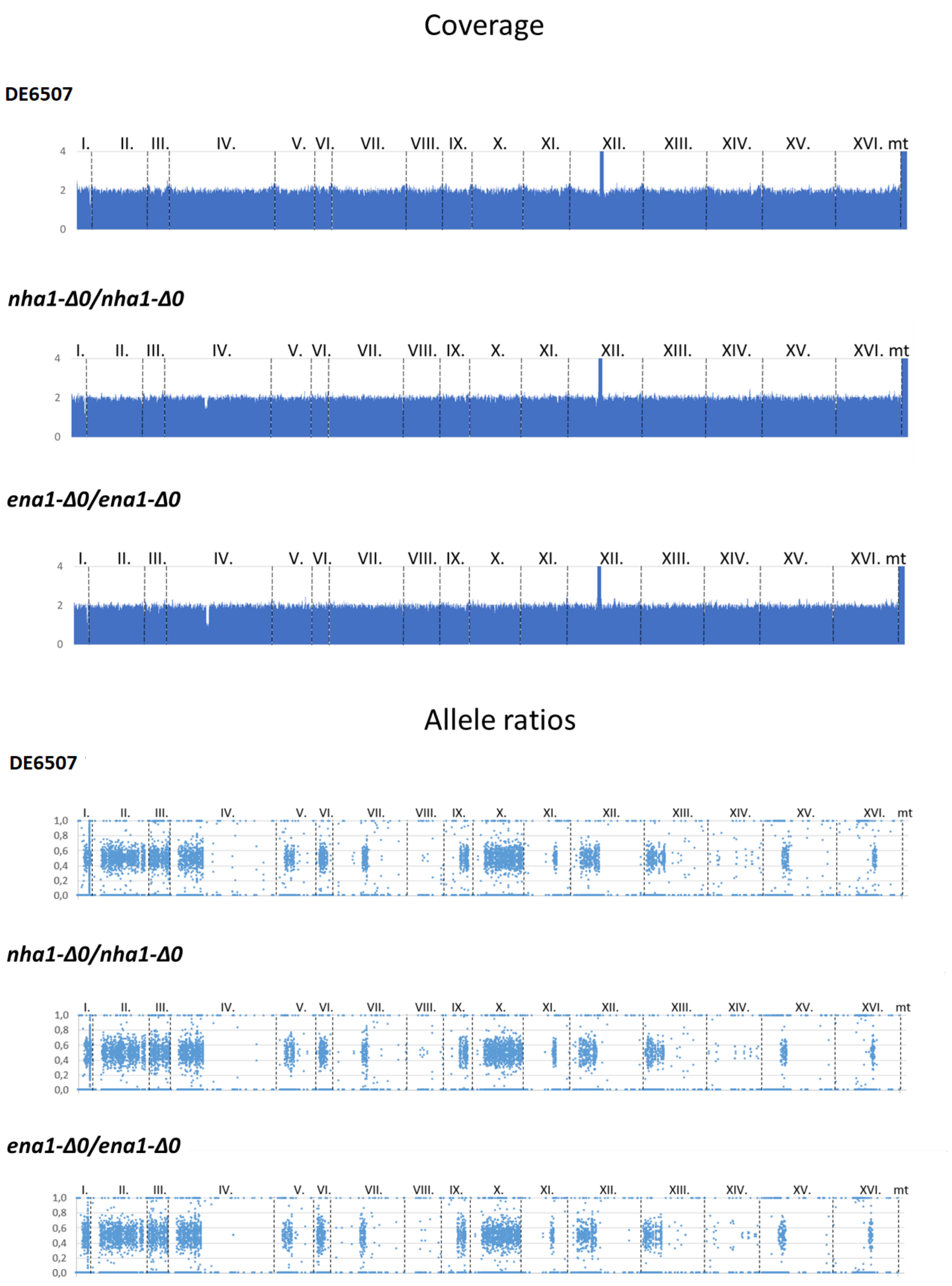

Figure S1e. Coverage and allele plots for DE6507 wild type isolate and the respective knockout strains. Sequencing coverage in 10 kb bins, corrected for ploidy (top parts of the composite images) and allele ratio plots (bottom parts) are all shown along the 16 chromosomes.

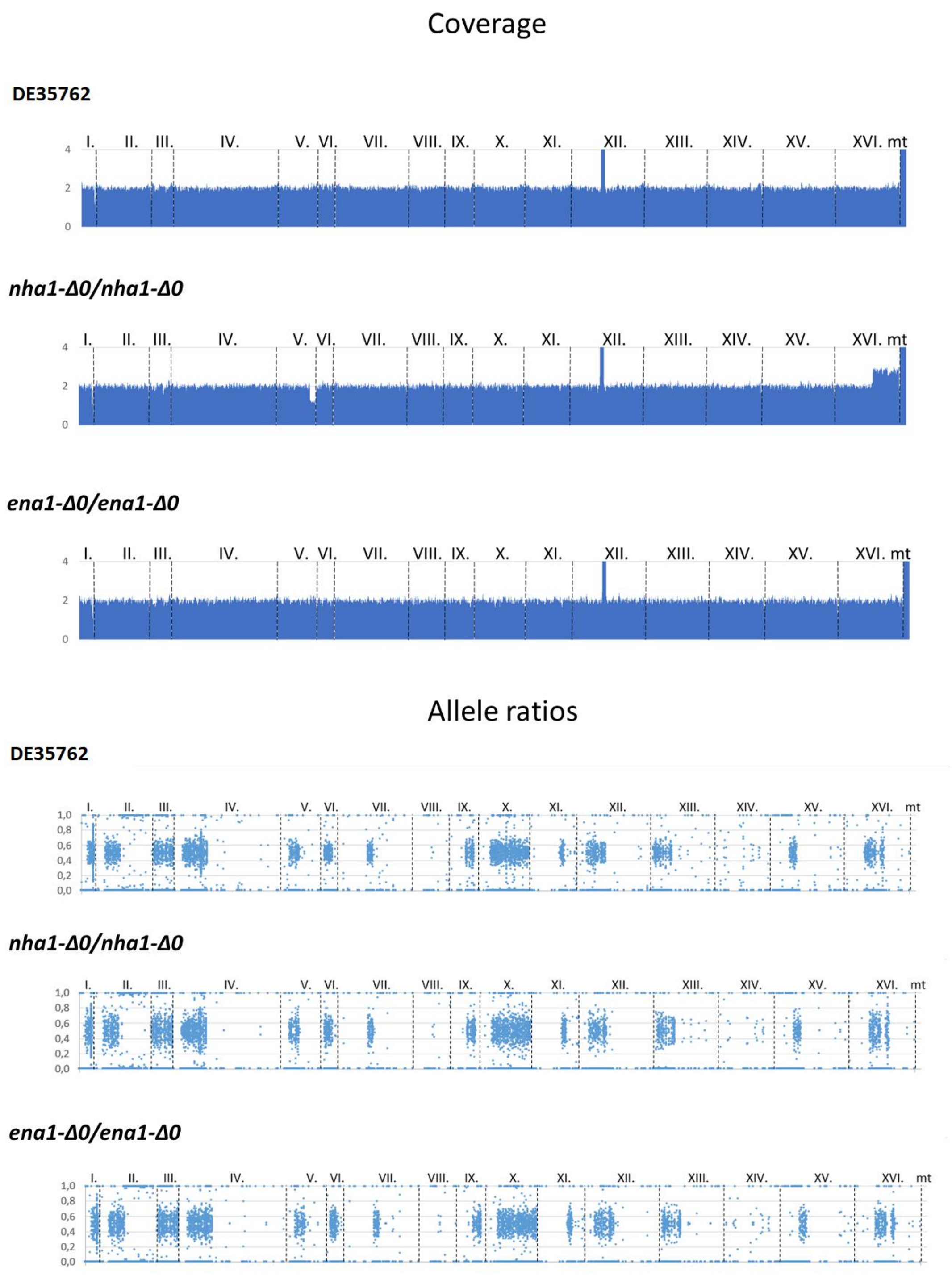

Figure S1f. Coverage and allele plots for DE35762 wild type isolate and the respective knockout strains. Sequencing coverage in 10 kb bins, corrected for ploidy (top parts of the composite images) and allele ratio plots (bottom parts) are all shown along the 16 chromosomes.

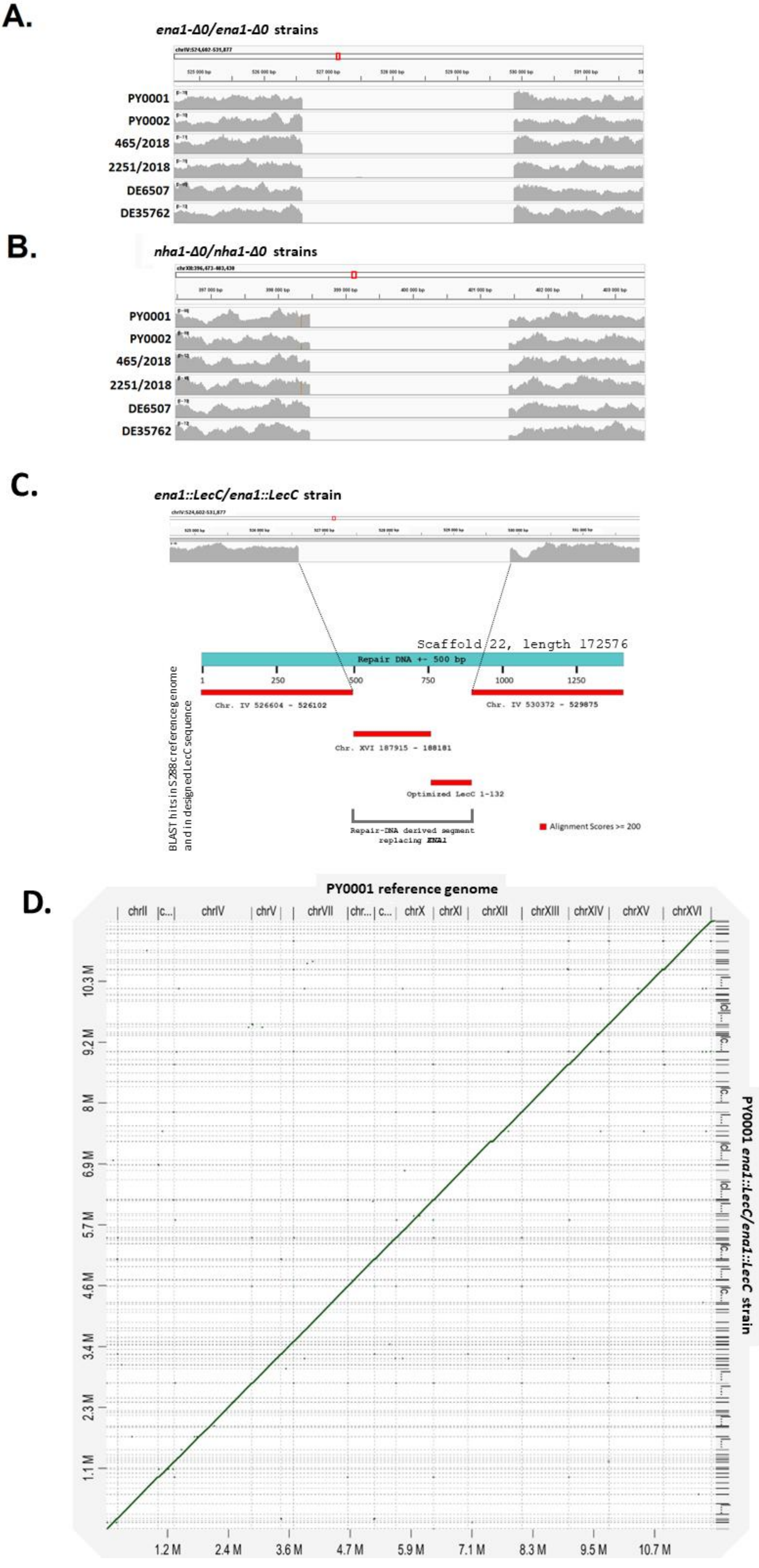
A.
ena1-Δ0/ena1-Δ0 strains
PY0001
PY0002
465/2018
2251/2018
DE6507
DE35762
B.
nha1-Δ0/nha1-Δ0 strains
PY0001
PY0002
465/2018
2251/2018
DE6507
DE35762
C.
ena1::LecC/ena1::LecC strain
Scaffold 22, length 172576
Repair DNA +- 500 bp
1
250
500
750
1000
1250
Chr. IV 526604 - 526102
Chr. IV 530372 - 529875
Chr. XVI 187915 - 188181
Optimized LecC 1-132
Repair-DNA derived segment replacing ENA1
Alignment Scores >= 200
BLAST hits in S288c reference genome and in designed LecC sequence
D.
PY0001 reference genome
chrII
chrIV
chrV
chrVII
chrX
chrXI
chrXII
chrXIII
chrXIV
chrXV
chrXVI
10.3 M
9.2 M
8 M
6.9 M
5.7 M
4.6 M
3.4 M
2.3 M
1.1 M
1.2 M
2.4 M
3.6 M
4.7 M
5.9 M
7.1 M
8.3 M
9.5 M
10.7 M
PY0001 ena1::LecC/ena1::LecC strain

Figure S2. Genomics results on the targeted loci. **A–B:** Per-base coverage analysis at the loci of targeted genes in the deletion strains. For each deletion strain, coverage at the locus of the target gene and 2000 bp up- and downstream regions are shown, minimum and maximum coverage values of mapped reads are indicated on the left edges of graphs. On the top, relative position on each chromosome is shown. **A:** coverage graphs in the case of *NHA1* gene deletion. **B:** coverage graphs in the case of *ENA1* gene deletion. **C:** coverage graphs in the case of *ENA1* gene deletion and an illustration of the correct integration of the LecC gene with a 5' α-mating factor secretion signal sequence (originally located on the chr. XVI of *S. cerevisiae*, including the PY0001 isolate). BLAST results on the identification of sequences in the scaffold containing the secretion signal and LecC repair DNA shown in the bottom part of the figure. All BLAST alignment scores were above 200, confirming the presence of the secretion signal directly followed by the LecC gene, replacing the *ENA1* gene. **D:** Pairwise alignment of the assembled PY0001 *ena1::LecC/ena1::LecC* strain's genome (vertical) and the PY0001 reference genome (horizontal).

	PY0001 knockout		PY0002 knockout		465/2018 knockout		2251/2018 knockout		DE6507 knockout		DE35762 knockout	
	nha1-Δ0/nha1-Δ0 **(non-norm.)**	***ena1-Δ0/ena1-Δ0*** **(normal)**	***nha1-Δ0/nha1-Δ0*** **(normal)**	***ena1-Δ0/ena1-Δ0*** **(normal)**	***nha1-Δ0/nha1-Δ0*** **(normal)**	***ena1-Δ0/ena1-Δ0*** **(normal)**	***nha1-Δ0/nha1-Δ0*** **(non-norm.)**	***ena1-Δ0/ena1-Δ0*** **(normal)**	***nha1-Δ0/nha1-Δ0*** **(non-norm.)**	***ena1-Δ0/ena1-Δ0*** **(normal)**	***nha1-Δ0/nha1-Δ0*** **(normal)**	***ena1-Δ0/ena1-Δ0*** **(normal)**
**PY0001 (normal)**	n.s. Mann-Whitney test	*** *p*= 0.000082 *ena1-Δ0/ena1-Δ0* > w.t Mann-Whitney test										
**PY0002 (normal)**			n.s. Mann-Whitney test	n.s. Mann-Whitney test								
**465/2018 (normal)**					n.s. Mann-Whitney test	* *p*=0.020524 w.t. > *ena1-Δ0/ena1-Δ0* Mann-Whitney test						
**2251/2018 (non-norm.)**							n.s. Mann-Whitney test	n.s. Mann-Whitney test				
**DE6507 (normal)**									n.s. Mann-Whitney test	** *p*=0.002838 *ena1-Δ0/ena1-Δ0* > w.t. Mann-Whitney test		
**DE35762 (non-norm.)**											** *p*=0.001257 *nha1-Δ0/nha1-Δ0* > w.t. Mann-Whitney test	** *p*=0.002422 *ena1-Δ0/ena1-Δ0* > w.t. Mann-Whitney test

Table S5. Statistical analysis of CFU/kidney weight (g) values of wild-type isolates and their corresponding knock-out strains. Normal or non-normal distribution of the recorded data indicated for all yeast strains. Chosen statistical test based on normality and variances is indicated. n.s.: non-significant; *: $p < 0.05$; **: $p < 0.01$; and ***: $p < 0.001$. w.t. = wild-type. Number of mice used for the experiments was 9 (PY0001, PY0002, DE6507,

DE35762), 8 (PY0001 *nha1-Δ0/nha1-Δ0*, PY0001 *ena1-Δ0/ena1-Δ0*, *PY0002 ena1-Δ0/ena1-Δ0*, 465/2018 *nha1-Δ0/nha1-Δ0*, 465/2018 *ena1-Δ0/ena1-Δ0,* 2251/2018 *nha1-Δ0/nha1-Δ0*, 2251/2018 *ena1-Δ0/ena1-Δ0*, DE6507 *nha1-Δ0/nha1-Δ0*, DE6507 *ena1-Δ0/ena1-Δ0*, DE35762 *nha1-Δ0/nha1-Δ0*, DE35762 *ena1-Δ0/ena1-Δ0*), and 7 (PY0002 *nha1-Δ0/nha1-Δ0*, 465/2018, 2251/2018). Wild-type and respecting knockout strains were compared.

	**PY0001** ***ena1-Δ0/ ena1-Δ0***	**PY0001** ***ena1::LecC/ ena1::LecC***	**PY0002** ***ena1-Δ0/ ena1-Δ0***	**465/2018** ***ena1-Δ0/ ena1-Δ0***	**2251/2018** ***ena1-Δ0/ ena1-Δ0***	**DE6507** ***ena1-Δ0/ ena1-Δ0***	**DE35762** ***ena1-Δ0/ ena1-Δ0***
**PY0001**	PY0001 *ena1-Δ0/ ena1-Δ0* > PY0001 * p=0.0134	PY0001 *ena1::LecC/ ena1::LecC* > PY0001 ** p=0.0031					
**PY0002**	n.d.	n.d.	PY0002 *ena1-Δ0/ ena1-Δ0* > PY0002 *** p=0.0005				
**465/2018**				465/2018 *ena1-Δ0/ ena1-Δ0* > 465/2018 * p=0.0246			
**2251/2018**					n.s. p=0.4880		
**DE6507**						n.s. p=0.1292	
**DE35762**							n.s. p=0.0628

Table S6. Statistical comparison of survival curves with Log-rank (Mantel-Cox) method in the 21-day infection experiments for each commercial or clinical isolate and for the respective knockout strains, using eight mice per test. n.s.: non-significant; *: $p < 0.05$; **: $p < 0.01$; and ***: $p < 0.001$. Number of mice used for the experiments was n=8 for each yeast. Wild-type and respecting knockout strains were compared.

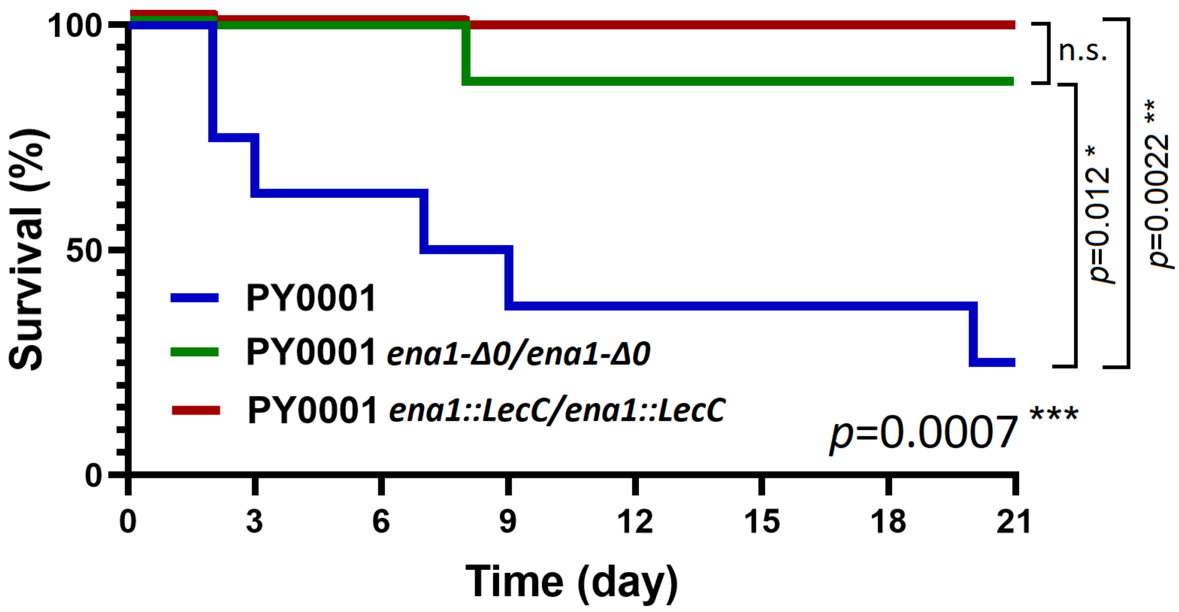

Figure S3. Survival curves of mice inoculated with PY0001 and its two modified strains in 21-day-long infection experiments. Kaplan-Meier survival curves and results of Log-rank (Mantel-Cox) test are shown, survival curves are slightly displaced when overlapping for clarity. n.s.: not significant; *: $p < 0.05$; **: $p < 0.01$; ***: $p < 0.001$. Number of mice used for the experiments was n=8 for each yeast.

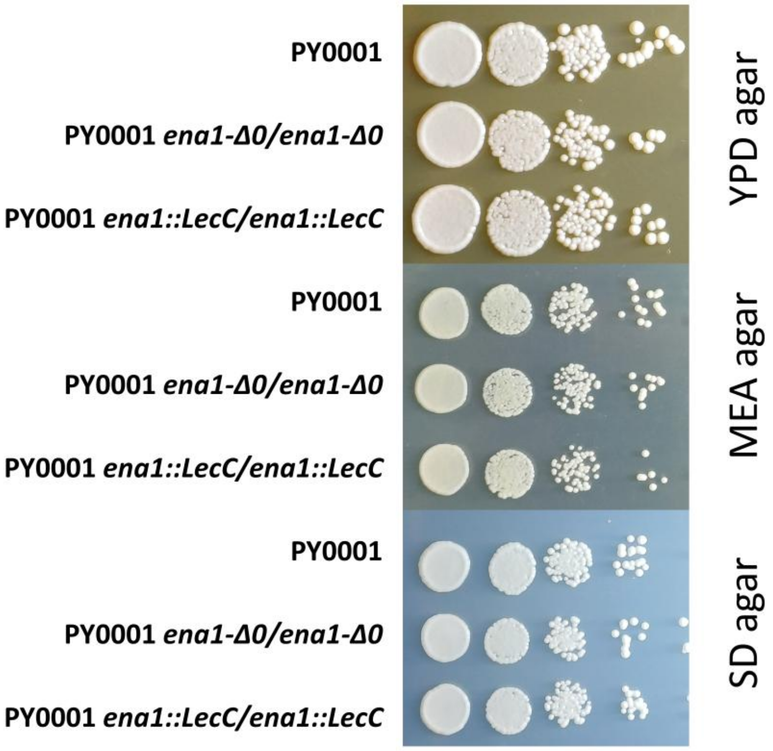

Figure S4. Spot plate assay with PY0001 and its two modified strains on three commonly used yeast media (YPDA, MEA, SDA) after 2 days of incubation at 37°C, illustrating that growth capabilities were not diminished. Plated spots contained ~10^4, 10^3, 10^2, and 10 cells.

**Growth score**	**YPD non-buffered**	**MEA**	**SDA**
**PY0001**	4	4	4
**PY0001** ***ena1-Δ0/ena1-Δ0***	4	4	4
**PY0001** ***ena1::LecC/ena1::LecC***	4	4	4

Table S7. Results of spot-plate assays with PY0001 and its modified strains on commonly used yeast media after 2 days of incubation at 37°C, illustrating that growth capabilities were not diminished.

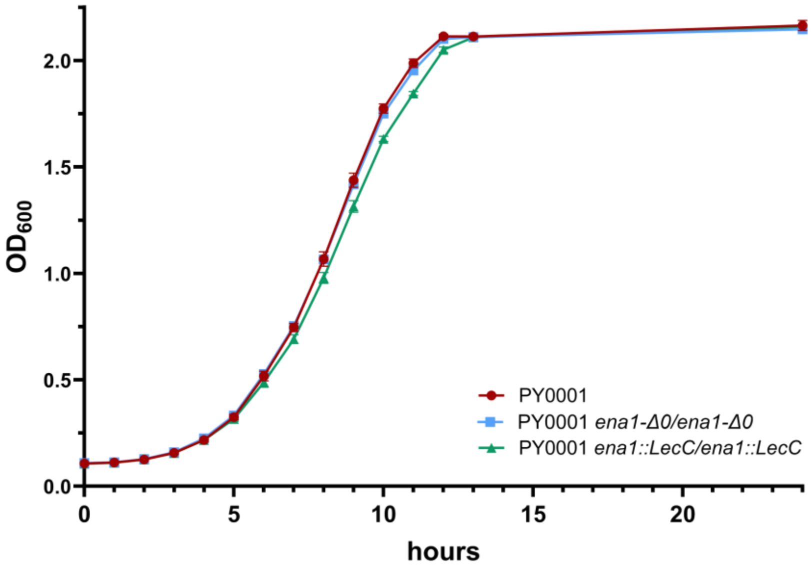

Figure S5. Growth curves of the PY0001 and its two modified strains. Error bars represent standard deviation of triplicate samples. Growth was followed by measuring OD at 600 nm for 13 hours, and at 24 hours of incubation, illustrating that growth capabilities of the modified strains closely match that of PY0001.

	**replicate 1**	**replicate 2**	**replicate 3**
**PY0001**	2.14 h	2.07 h	2.13 h
**PY0001** ***ena1-Δ0/ena1-Δ0***	2.17 h	2.15 h	2.25 h
**PY0001** ***ena1::LecC/ena1::LecC***	2.14 h	2.13 h	2.20 h
**significant differences**	No significant difference was found between the samples (one-way ANOVA)		

Table S8. Doubling times in YPD medium at 37°C of the PY0001 and its two modified strains in triplicates, calculated from the OD values between 7 h and 9 h of the experiment depicted in Figure S4, rounded to two decimals.

	**replicate 1**	**replicate 2**	**replicate 3**
**PY0001**	0.135 g	0.16 g	0.177 g
**PY0001** ***ena1-Δ0/ena1-Δ0***	0.13 g	0.165 g	0.136 g
**PY0001** ***ena1::LecC/ena1::LecC***	0.172 g	0.148 g	0.159 g
**significant differences**	No significant difference was found between the samples (one-way ANOVA)		

Table S9. Dry biomass production of the wild type isolate PY0001 and its two mutants in 100 mL YPD medium at 37°C in 24 h, from the experiment depicted in Figure S4.

	replicate 1	replicate 2	replicate 3
**PY0001**	3.14%	3.56%	3.22%
**PY0001**<br>***ena1-Δ0/ena1-Δ0***	1.67%	1.80%	1.40%
**PY0001**<br>***ena1::LecC/ena1::LecC***	5.0 %	4.68%	4.68%
**significant differences**	$p < 0.0001$ ****<br>PY0001 > PY0001 *ena1-Δ0/ena1-Δ0* $p < 0.001$ ***<br>PY0001 *ena1::LecC/ena1::LecC* > PY0001 $p < 0.001$ ***<br>PY0001 *ena1::LecC/ena1::LecC* > PY0001 *ena1-Δ0/ena1-Δ0* $p < 0.0001$ ****<br>(one-way ANOVA)		

Table S10. Survival percentage of cells of PY0001 and its two modified strains in triplicates after liophilization. ***: $p < 0.001$; ****: $p < 0.0001$.

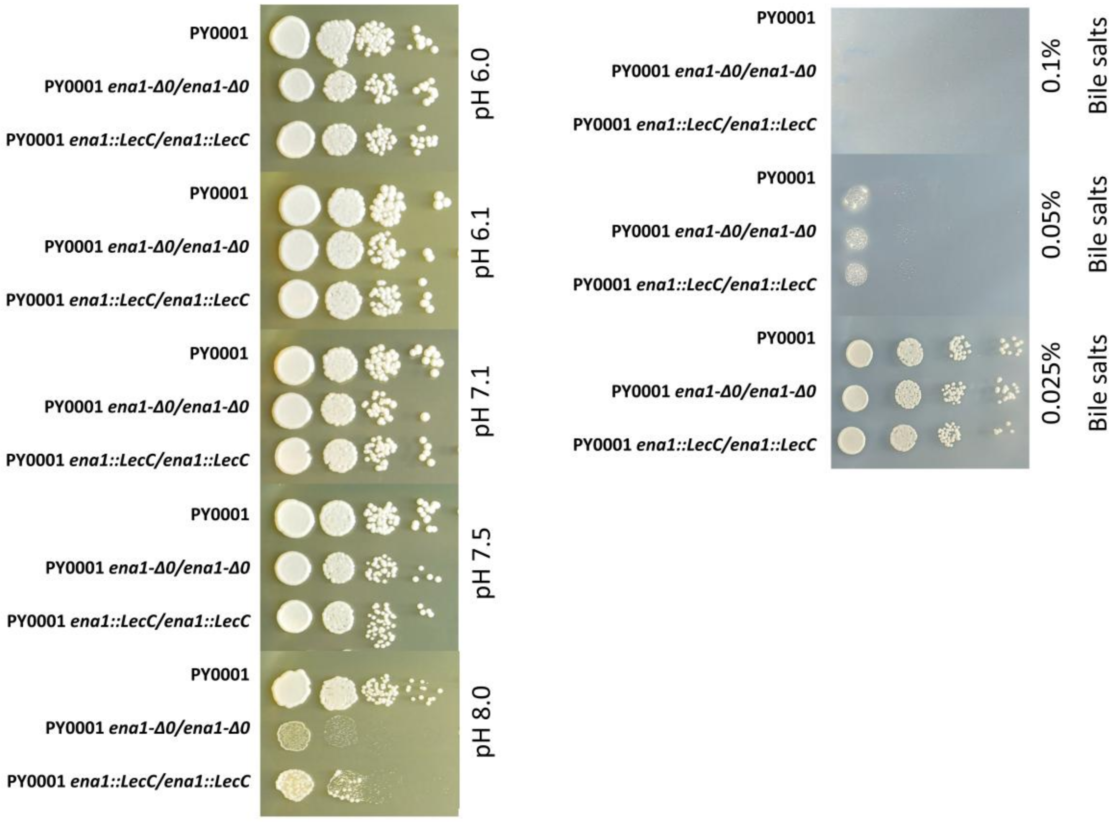

Figure S6. Spot plate assay with PY0001 and its two modified strains on YPD medium buffered to various physiologically relevant pH values or supplemented with bile salts (w/v % shown) after 2 days of incubation at 37°C. Plated spots contained ~10^4, 10^3, 10^2, and 10 cells.

Tolerance score	YPD pH 6.0	YPD pH 6.1	YPD pH 7.1	YPD pH 7.5	YPD pH 8.0	Bile salts 0.1%	Bile salts 0.5%	Bile salts 0.025%
**PY0001**	4	4	4	4	4	0	1	4
**PY0001 *ena1-Δ0/ena1-Δ0***	4	4	4	4	1	0	1	4
**PY0001 *ena1::LecC/ena1::LecC***	4	4	4	4	2	0	1	4

Table S11. Results of spot-plate assays with PY0001 and its modified strains on YPD buffered to various pH values, and on bile acid containing SD agar plates.

	Amphotericin B (0.03-16 mg/L)	Fluconazole (0.125-64 mg/L)	Anidulafungin (0.004-2 mg/L)	Caspofungin (0.004-2 mg/L)	Micafungin (0.004-2 mg/L)
**PY0001**	1	2	0.25	0.004	0.5
**PY0001 *ena1-Δ0/ena1-Δ0***	1	0.5	0.25	0.06	1
**PY0001 *ena1::LecC/ena1::LecC***	0.5	0.5	0.25	0.03	1

Table S12. MIC values (mg/L) determined for PY0001 and its modified strains for five antifungal agents. For each antifungal, the applied concentrations in the antifungal test panel are given in parentheses. Following the CLSI protocol, samples were tested in a single experiment.

	Antagonism test with culture supernatant					
**species**	***Bacillus subtilis***			***E. coli***		
**note**	**complete inhibition**			**incomplete inhibition**		
**replicate**	**replicate 1**	**replicate 2**	**replicate 3**	**replicate 1**	**replicate 2**	**replicate 3**
**PY0001**	13.2 mm	12.7 mm	12.5 mm	19.2 mm	19.8 mm	19.6 mm
**PY0001 *ena1-Δ0/ena1-Δ0***	13.7 mm	12.8 mm	12.9 mm	19.7 mm	19.3 mm	19.5 mm
**PY0001 *ena1::LecC/ena1::LecC***	14.1 mm	12.7 mm	12.7 mm	18.7 mm	18.1 mm	19.4 mm
**significant differences**	No significant difference was found between the samples (one-way ANOVA)			No significant difference was found between the samples (one-way ANOVA)		
	**Antagonism test with culture supernatant**					
**species**	***K. oxytoca*** **group**			***P. putida*** **group**		
**note**	**incomplete inhibition**			**incomplete inhibition**		
**replicate**	**replicate 1**	**replicate 2**	**replicate 3**	**replicate 1**	**replicate 2**	**replicate 3**
**PY0001**	17.5 mm	17.8 mm	17.4 mm	18.2 mm	18.5 mm	18.6 mm
**PY0001 *ena1-Δ0/ena1-Δ0***	17.4 mm	18.2 mm	17.2 mm	18.3 mm	18.9 mm	18.3 mm
**PY0001 *ena1::LecC/ena1::LecC***	17.1 mm	18.2 mm	17.3 mm	18.3 mm	18.0 mm	18.2 mm
**significant differences**	No significant difference was found between the samples (one-way ANOVA)			No significant difference was found between the samples (one-way ANOVA)		
	**Antagonism test with culture supernatant**			**Antagonism test with concentrated dialysed supernatant**		
**species**	***L. monocytogenes***			***L. monocytogenes***		
**note**	**no inhibition**			**complete inhibition**		
**replicate**	**replicate 1**	**replicate 2**	**replicate 3**	**replicate 1**	**replicate 2**	**replicate 3**
**PY0001**	0 mm	0 mm	0 mm	0 mm	0 mm	0 mm
**PY0001 *ena1-Δ0/ena1-Δ0***	0 mm	0 mm	0 mm	0 mm	0 mm	0 mm
**PY0001 *ena1::LecC/ena1::LecC***	0 mm	0 mm	0 mm	11.6 mm	12.3 mm	11.5 mm
**significant differences**	No significant difference was found between the samples (one-way ANOVA)			$p < 0.0001$ ****<br>PY0001ΔΔ*ENA1*LecC > PY0001<br>$p < 0.0001$ ****<br>PY0001ΔΔ*ENA1*LecC > PY0001ΔΔ*ENA1*<br>$p < 0.0001$ ****<br>(one-way ANOVA)		

Table S13. Inhibition zones of the supernatants and concentrated supernatants of PY0001 and its modified strains in agar well diffusion assays against various bacteria. Results of 3 replicate antagonism tests are compared using on one-way ANOVA and Tukey HSD, ****: $p < 0.0001$. In the case of supernatants with a pH set to 6.0, inhibition zones did not appear for any of the samples and hence, no statistical comparison was made.

	replicate 1	replicate 2	replicate 3
**PY0001**	3.08%	4.92%	3.08%
**PY0001 *ena1-Δ0/ena1-Δ0***	6.80%	6.40%	4.00%
**PY0001 *ena1::LecC/ena1::LecC***	3.00 %	2.50%	4.50%
**significant differences**		No significant difference was found between the samples (one-way ANOVA)	

Table S14. Survival of PY0001 and its two modified strains under simulated gastrointestinal conditions (simulated salivary fluid, gastric fluid, and intestinal fluid) in percentage of total cell number at the start of the experiment in 3 replicates.

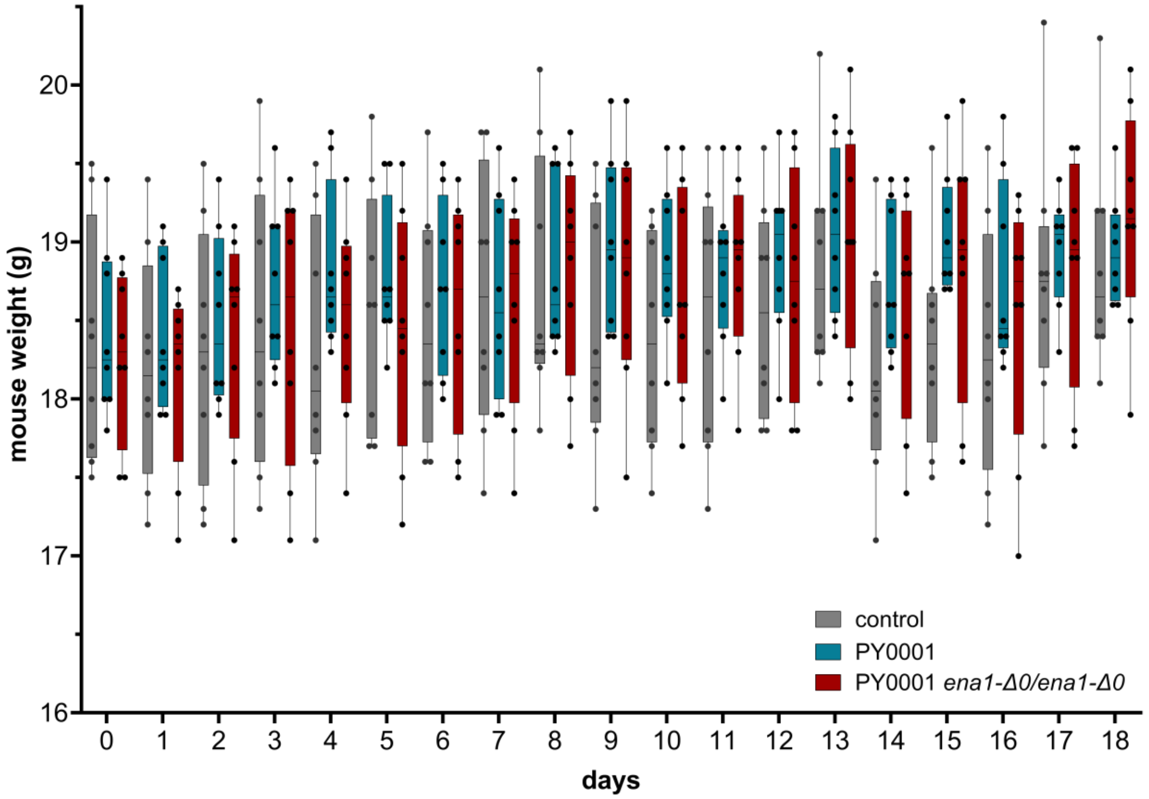

Figure S7. Weight of the mice in the control group and in the yeast gavaged groups (PY0001 and PY0001 *ena1-Δ0/ena1-Δ0*) during gavaging experiments over the 14 days of the experiment and 4 days of post-gavaging period. A data point represents the weight (g) of an individual mouse on a particular day. Horizontal black lines represent the median of the data points, and whiskers extend to minimum and maximum values. No significant difference in mouse weight was found among groups (two-way ANOVA). Each group contained n=8 mice, all survived the experimental period.

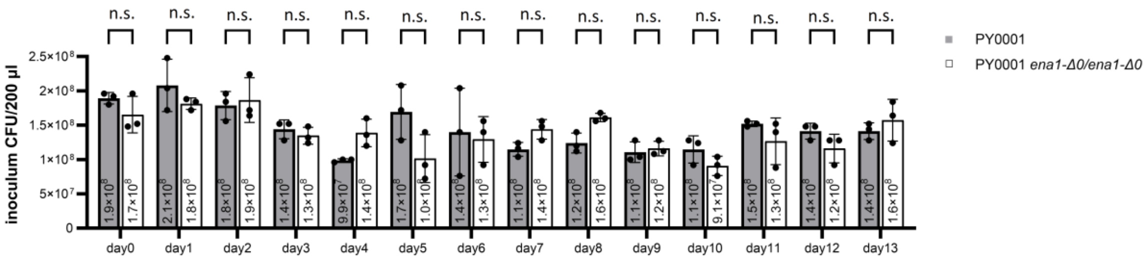

Figure S8. CFU numbers for inocula prepared and used on each day of the gavaging experiments, shown for the 200 µL volumes that were gavaged. On all days of the experiment, the inocula of the two yeast strains (PY0001 and PY0001 *ena1-Δ0/ena1-Δ0*) contained cell densities that were not statistically different. Each inoculum's cell density was determined in triplicates (n=3 technical replicates). Bars show the mean, whiskers show the standard deviation of the data. Unpaired T-test was applied for each pair of data. n.s.: not significant.

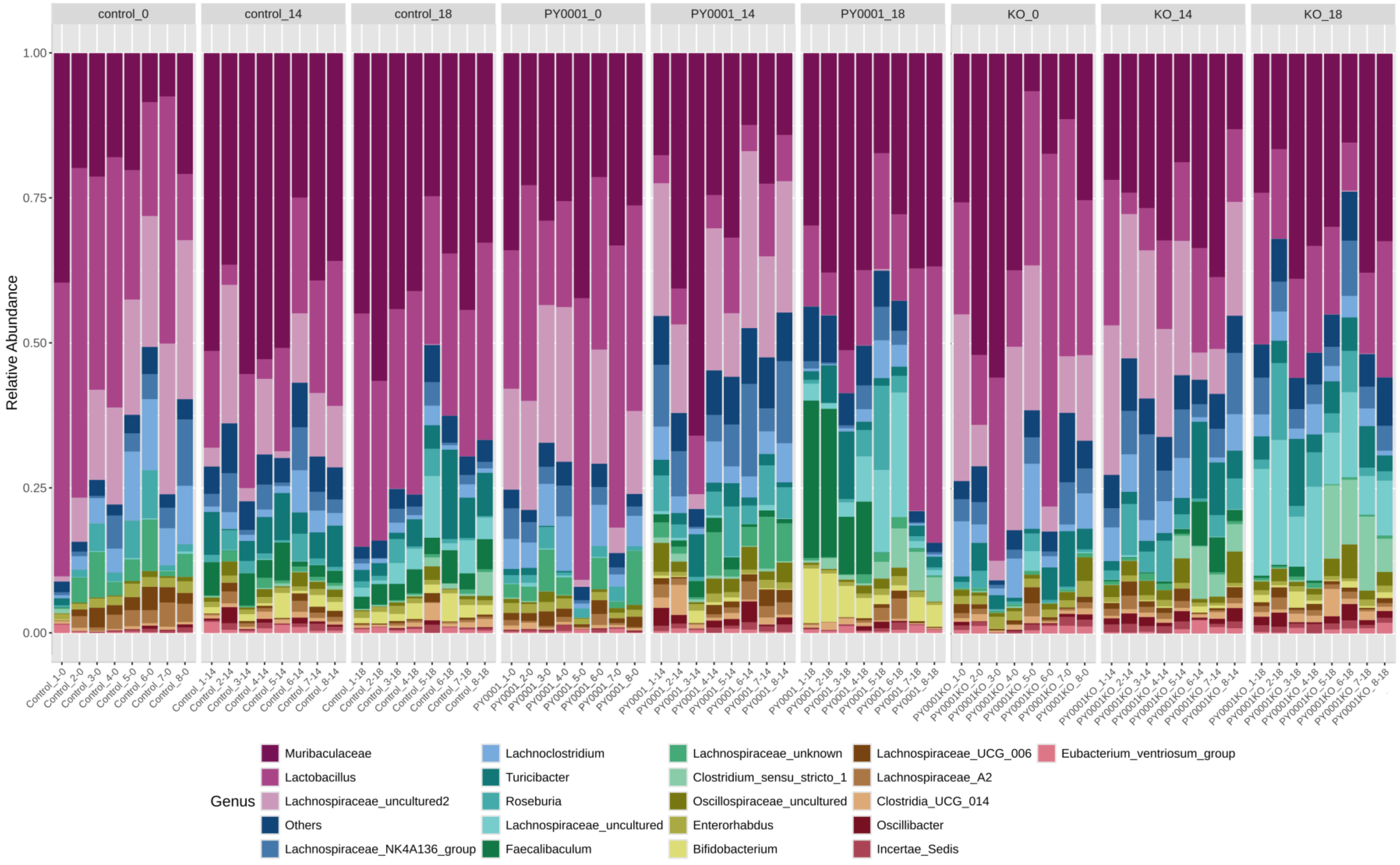

Figure S9. Relative bacterial abundance data based on 16S-sequencing for individual mice of the control group and the probiotic yeast treated groups, PY0001 and PY0001 *ena1-Δ0/ena1-Δ0* (latter shortened as PY0001KO on the image). Fecal samples were collected before the start of the experiment (0 d), 14th day of treatment (14 d) and four days after the stop of gavaging (18 d). Genus-level groups of bacteria are shown with their relative abundance, color-coded. The top 20 most abundant taxa are shown and less abundant taxa are merged as “Others”. Incertae Sedis refers to an unplaced Ruminococcaceae taxon. Each sample’s name refer to the strain, mouse identification number (1 to 8) and time in days (0, 14, and 18) on the x axis. Each mouse group contained 8 animals.

**Type of alpha diversity comparison**	**Compared groups**	**FDR-adjusted *p*-value**
groups at the start of the experiment	control day 0 *vs* PY0001 day 0	0.075558 n.s.
	control day 0 *vs* PY0001 *ena1-Δ0/ena1-Δ0* day 0	0.030707 * control day 0 < PY0001 *ena1-Δ0/ena1-Δ0* day 0
	PY0001 day 0 *vs* PY0001 *ena1-Δ0/ena1-Δ0* 0 day	0.53081 n.s.
groups at the end of gavaging	control day 14 *vs* PY0001 day 14	0.70606 n.s.
	control day 14 *vs* PY0001 *ena1-Δ0/ena1-Δ0* day14	0.70606 n.s.
	PY0001 day 14 *vs* PY0001 *ena1-Δ0/ena1-Δ0* day14	0.41914 n.s.
groups at the end of experiment	control day 18 *vs* PY0001 day 18	0.81727 n.s.
	control day 18 *vs* PY0001 *ena1-Δ0/ena1-Δ0* day 18	0.047658 * control day 18 < PY0001 *ena1-Δ0/ena1-Δ0* day 18
	PY0001 day 18 *vs* PY0001 *ena1-Δ0/ena1-Δ0* day 18	0.083921 n.s.
start vs. end of gavaging in each group	control day 0 *vs* control day 14	0.001167 ** control day 0 < control day 14
	PY0001 day 0 *vs* PY0001 day 14	0.001167 ** PY0001 day 0 < PY0001 day 14
	PY0001 *ena1-Δ0/ena1-Δ0* day 0 *vs* PY0001 *ena1-Δ0/ena1-Δ0* day 14	0.0023243 ** PY0001 *ena1-Δ0/ena1-Δ0* day 0 < PY0001 *ena1-Δ0/ena1-Δ0* day 14
end of gavaging vs. end of experiment in each group	control day 14 *vs* control day 18	0.0018824 ** control day 14 > control day 18
	PY0001 day 14 *vs* PY0001 day 18	0.0018922 ** PY0001 day 14 > PY0001 day 18
	PY0001 *ena1-Δ0/ena1-Δ0* day 14 *vs* PY0001 *ena1-Δ0/ena1-Δ0* day 18	0.027836 * PY0001 *ena1-Δ0/ena1-Δ0* day 14 > PY0001 *ena1-Δ0/ena1-Δ0* day 18
start vs. end of experiment in each group	control day 0 *vs* control day 18	0.13033 n.s.
	PY0001 day 0 *vs* PY0001 day 18	1 n.s.
	PY0001 *ena1-Δ0/ena1-Δ0* day 0 *vs* PY0001 *ena1-Δ0/ena1-Δ0* day 18	0.1433 n.s.

Table S15. Results of bacterial alpha-diversity comparisons in the fecal samples of mice gavaged with PY0001 and PY0001 *ena1-Δ0/ena1-Δ0* and control animals on day 0 and 14 of the experiment. Filtered bacterial abundance data was subjected to alpha diversity calculation based on genera, with Chao1 diversity measure, and ANOVA with Welch's T-test post-hoc pairwise comparisons. Multi-testing adjustment is based on Benjamini-Hochberg procedure, all conducted in MicrobiomeAnalyst 2.0. *p*-value for the ANOVA test of all samples was *p*=3.716e-11, in the table, only relevant comparisons are detailed. n.s.: non-significant; *: $p < 0.05$; **: $p < 0.01$. FDR: false discovery rate.

Type of beta diversity comparison	Compared groups	FDR-adjusted *p*-value
groups at the start of the experiment	control day 0 *vs* PY0001 day 0	0.32811 n.s.
	control day 0 *vs* PY0001 *ena1-Δ0/ena1-Δ0* day 0	0.17564 n.s.
	PY0001 day 0 *vs* PY0001 *ena1-Δ0/ena1-Δ0* 0 day	0.393 n.s.
groups at the end of gavaging	control day 14 *vs* PY0001 day 14	0.025714 *
	control day 14 *vs* PY0001 *ena1-Δ0/ena1-Δ0* day14	0.024 *
	PY0001 day 14 *vs* PY0001 *ena1-Δ0/ena1-Δ0* day14	0.32811 n.s.
groups at the end of experiment	control day 18 *vs* PY0001 day 18	0.0855 n.s.
	control day 18 *vs* PY0001 *ena1-Δ0/ena1-Δ0* day 18	0.0025714 **
	PY0001 day 18 *vs* PY0001 *ena1-Δ0/ena1-Δ0* day 18	0.0276 *
start vs. end of gavaging in each group	control day 0 *vs* control day 14	0.0036**
	PY0001 day 0 *vs* PY0001 day 14	0.0051429 **
	PY0001 *ena1-Δ0/ena1-Δ0* day 0 *vs* PY0001 *ena1-Δ0/ena1-Δ0* day 14	0.032516 *
end of gavaging vs. end of experiment in each group	control day 14 *vs* control day 18	0.0025714 **
	PY0001 day 14 *vs* PY0001 day 18	0.0036 **
	PY0001 *ena1-Δ0/ena1-Δ0* day 14 *vs* PY0001 *ena1-Δ0/ena1-Δ0* day 18	0.0025714 **
start vs. end of experiment in each group	control day 0 *vs* control day 18	0.0025714 **
	PY0001 day 0 *vs* PY0001 day 18	0.0036 **
	PY0001 *ena1-Δ0/ena1-Δ0* day 0 *vs* PY0001 *ena1-Δ0/ena1-Δ0* day 18	0.0025714 **

Table S16. Results of bacterial beta-diversity comparisons in the fecal samples of mice gavaged with PY0001 and PY0001 *ena1-Δ0/ena1-Δ0* and control animals on day 0 and 14 of the experiment. Filtered bacterial abundance data was subjected to beta diversity calculation based on genera, using Bray-Curtis distance index and pairwise PERMANOVA for comparisons. Multi-testing adjustment is based on Benjamini-Hochberg procedure, all conducted in MicrobiomeAnalyst 2.0. p-value for the PERMANOVA test of all samples was p=0.001, in the table, only relevant comparisons are detailed. n.s.: non-significant; *: $p < 0.05$; **: $p < 0.01$. FDR: false discovery rate.

	oligonucleotides targeting *ENA1*	oligonucleotides targeting *NHA1*
**guides**	forward: 5’- gactTTAAAGAAGAAGAAACCTCTGT-3’<br>reverse: 5’- aaacACAGAGGTTTCTTCTTCTTTAA-3’	forward: 5’-gactTTAGAAGAAGATGAGTACACAC-3’<br>reverse: 5’-aaacGTGTGTACTCATCTTCTTCTAA-3’
**repair DNA**	forward:<br>5’-AACAATACGTATATTTACTAATTAAAGAAAAAACTTCGTACACAGAATTGAAAATTTTCGcactaactaactaagcgtcg-3’<br><br>reverse:<br>5’-TAAGGGGGAGAAGGGATAAGGGATGCAAAAGGAAAGGCACTCAATAAATTGCCCTCCTTAcgacgcttagttagttagtg-3’	forward:<br>5’-TCCCAAGCATCGTGTTTTTTTGTACATTATAAAAAAAAATCCTGAACTTAGCTAGATATTcactaactaactaagcgtcg-3’<br><br>reverse:<br>5’-AAAAAGGCATTTCGTTTATATATATACTAAAATAATATATCTTTGTGTATTAATAAATTAcgacgcttagttagttagtg-3’
**verification**	Primers for *ENA1* gene amplification:<br>forward: 5’-ATCAAGTGAACTGTTCACAC-3’<br>reverse: 5’-GGGGTTGTGGTATTGATAGA-3’<br><br>Primers for rDNA cassette amplification:<br>forward: 5’-ATCAAGTGAACTGTTCACAC-3’<br>reverse: 5’-CGACGCTTAGTTAGTTAGTG-3’	Primers for *NHA1* gene amplification:<br>forward: 5’-AGCGGACTTAGCGATTTACATAGG-3’<br>reverse: 5’-GCCGCGGATTTTTGAACAATG-3’<br><br>Primers for rDNA cassette amplification:<br>forward: 5’-AGCGGACTTAGCGATTTACATAGG-3’<br>reverse: 5’-CGACGCTTAGTTAGTTAGTG-3’

	oligonucleotides targeting *ENA1* with LeucocinC gene integration
**guides**	as above
**repair DNA**	primers for LecC integrative cassette amplification<br>forward: 5’- AACAATACGTATATTTACTAATTAAAGAAAAAACTTCGTACACAGAATTGAAAATTTTCGatgagatttccttcaatttttactgc -3’<br>reverse: 5’-TAAGGGGGAGAAGGGATAAGGGATGCAAAAGGAAAGGCACTCAATAAATTGCCCTCCTTAttagttgtgccaaccagcgttacc -3’<br>Synthetic **LecC sequence** with upstream **α-mating factor secretion signal sequence** (color-coded):<br>5’-<br>ATGAGATTTCCTTCAATTTTTACTGCAGTTTTATTCGCAGCATCCTCCGCATTAGCTGCTCCAGTCAACACTACAACAGAAGATGAAACGGCACAAATTCCGGCTGAAGCTGTCATCGGTTACTTAGATTTAGAAGGGGATTTCGATGTTGCTGTTTTGCCATTTTCCAACAGCACAAATAACGGGTTATTGTTTATAAATACTACTATTGCCAGCATTGCTGCTAAAGAAGAAGGGGTATCTTTGGATAAAAGAGAGGCTGAAGCTAAAAACTACGGTAATGGTGTCCACTGTACCAAGAAGGGTTGTTCCGTTGACTGGGGTTACGCTTGGACTAACATCGCCAACAACTCTGTTATGAACGGTTTGACTGGTGGTAACGCTGGTTGGCACAACTAA -3’
**verification**	Primers for *ENA1* gene amplification: as above for oligonucleotides targeting *ENA1*

Table S17. Oligonucleotides used for genome editing and for verification. For repair oligonucleotides, annealing region is shown in lowercase letters.

**Genomics: sequencies, mappong, allele calling**	For Illumina sequencing, DNA isolation followed Hanna and Xiao[1]. The Illumina FASTQ sequencing files were trimmed and filtered using fastp for further analysis[2]. Mapping to the PY0001 reference genome (accession no. ASM2473226v1) was performed using the mem option of BWA 0.7.17[3]. Sorted BAM files were obtained using Samtools 1.7.[4] and Picard-tools 2.23.8. was used to mark duplicated reads[5]. We used BEDTools 2.30.0[6] to calculate the median coverage of chromosomes in 10000 base windows sliding every 5000 bases. Plots generated from this data were corrected for ploidy and was used identify potential segmental duplications, deletions, or aneuploidies. Coverage was also visualized and compared on a per-base basis using IGV[7]. Using BAM files, local realignment around indels and joint variant calling and filtering for the strains and isolates were performed with GATK 4.1.9.0.[5,8] with regions annotated in the PY0001 reference as centromeric regions, telomeric regions, or LTRs excluded. First, genomic VCF files were obtained with the Haplotype Caller, and joint genotyping of the gVCF files was applied. After joint calling, in the resulting VCF files, only SNPs or only INDELS were selected. SNPs were filtered according to the parameters[9]: QD < 5.0; QUAL < 30.0; SOR > 3.0; FS > 60.0; MQ < 40.0; MQRankSum < -12.5; ReadPosRankSum < -8.0. INDELS were filtered according to the parameters QD < 5.0; QUAL < 30.0; FS > 60.0; ReadPosRankSum < -20.0. INDELS were then left-aligned. For the final VCF files, INDELS and SNPs were merged, filtered and non-variant sites were removed. Combined called VCF files were uploaded to FigShare (doi: 10.6084/m9.figshare.27105919).
**Comparative genomics**	Variants, their allelic depth, and called genotyes in the individual strains were selected and exported to a .csv file using the query option of SAMtools/BCFtools 1.10.2. Allele frequency plots were obtained by calculating the fraction of the two alleles' dept at ach heterozygous site. Allele frequencies were used to verify ploidy, with the assumptions that disomic chromosomes have allele ratios of approx. 1:0 or 1:1, trisomic of 1:0, 1:2, and 2:1, tetrasomic of 1:0, 1:3, 1:1, or 3:1, etc.[10–12]. Genotype calls were used to determine levels of heterozygosity for each chromosome of the various sequenced genomes, these values were then compared.
**Draft assembly**	For a draft assembly of the LecC-integrated PY0001 strain, we used Spades 3.12.0[13] with default settings on the fastp-trimmed Illumina reads. The draft assembly produced 1127 scaffolds amounting to 11,655,577 bases, of these 44 larger than 100,000 bp. An NCBI BLAST search was used to identify the yeast-optimized LecC gene, the α-mating factor secretion signal sequence, and the regions around the knocked-out *ENA1* gene in the assembled PY0001 genome. Sequences not aligned to the reference PY0001 genome were removed based on an alignment performed using D-GENIES[14]. The cleaned assembly was again aligned and visualized. The assembly is deposited in GenBank, accession number SUB15769654.

**Bacterial metabarcoding**	In the frames of the paid metabarcoding analysis at SeqCenter, sequences were imported to Qiime2 for analysis. Primer sequences were removed using Qiime2's[15] cutadapt2 plugin using the following degenerate primer queries: CCTAYGGGNBGCWGCAG (forward) and GACTACNVGGGTMTCTAATCC (reverse). Sequences were then denoised using Qiime2's dada2 plugin[16]. Denoised sequences were placed into a feature table detailing which amplicon sequence variants (ASVs) were observed in which samples, and how many times each ASV was observed in each sample. The ASVs were identified taxonomically using the Silva 138 99% full-length sequence database with the VSEARCH[17] utility in Qiime2's feature-classifier plugin. ASVs were then collapsed to their lowest taxonomic units.
**Metabarcoding data analysis**	Further analysis of the results provided by the company were carried out in-house using MicrobiomeAnalyst 2.0[18]. First, a taxonomy file was created for the abundance data and unclear higher systematic status were manually edited to replace multiple identical entries like "uncultured_bacterium" shared by unrelated species with unique ones. All genus-level names were changed to be unique, e.g. an uncultured_bacterium entry in Lachnospiraceae was changed to Lachnospiraceae_uncultured. An abundance table was also created with the modified species-level names. Then MicrobiomeAnalyst was used to filter data (low count filter; minimum count 4 in in at least 20% of occurrences) and to apply total sum scaling. Low-variance taxa were not filtered out. Stacked bar charts with relative frequency data were created for each sample, then all taxa's relative frequencies in each group and at each timepoint was also averaged for an overview image. Alpha diversity was assessed using filtered data for genera, using Chao1 diversity measure (total richness), with comparisons by ANOVA with posthoc pairwise comparisons. Beta diversity was also assessed for the genera, with PCoA method, Bray-Curtis distance index, using pairwise PERMANOVA statistics.

Table S18. Methods and software used in genomic and metabarcoding analysis.

	BioSample	SRA experiment	SRA run	Reference
**PY0001**	SAMN26521241	SRX14794981	SRR18693990	[11]
**PY0001** ***nha1-Δ0/nha1-Δ0***	SAMN43920984	SRX26194606	SRR30793137	this study
**PY0001** ***ena1-Δ0/ena1-Δ0***	SAMN43920985	SRX26194607	SRR30793136	this study
**PY0001** ***ena1::LecC/ena1::LecC***	SAMN53206793	SRX31058057	SRR36008767	this study
**PY0002**	SAMN26521565	SRX14795022	SRR18694027	[11]
**PY0002** ***nha1-Δ0/nha1-Δ0***	SAMN43920986	SRX26194610	SRR30793132	this study
**PY0002** ***ena1-Δ0/ena1-Δ0***	SAMN43920987	SRX26194611	SRR30793133	this study
**465/2018**	SAMN26521575	SRX14795021	SRR18694028	[11]
**465/2018** ***nha1-Δ0/nha1-Δ0***	SAMN43920988	SRX26194612	SRR30793130	this study
**465/2018** ***ena1-Δ0/ena1-Δ0***	SAMN43920989	SRX26194613	SRR30793131	this study
**2251/2018**	SAMN26521576	SRX14795020	SRR18694029	[11]
**2251/2018** ***nha1-Δ0/nha1-Δ0***	SAMN43920990	SRX26194614	SRR30793129	this study
**2251/2018** ***ena1-Δ0/ena1-Δ0***	SAMN43920991	SRX26194615	SRR30793128	this study
**DE6507**	SAMN27484089	SRX14795019	SRR18694030	[11]
**DE6507** ***nha1-Δ0/nha1-Δ0***	SAMN43920992	SRX26194616	SRR30793127	this study
**DE6507** ***ena1-Δ0/ena1-Δ0***	SAMN43920993	SRX26194617	SRR30793126	this study
**DE35762**	SAMN27488932	SRX14795018	SRR18694031	[11]
**DE35762** ***nha1-Δ0/nha1-Δ0***	SAMN43920994	SRX26194608	SRR30793135	this study
**DE35762** ***ena1-Δ0/ena1-Δ0***	SAMN43920995	SRX26194609	SRR30793134	this study

Table S19. Metadata of the isolates' and modified strains' whole genome sequencing data.

Supplementary references

1. Hanna, M. & Xiao, W. Isolation of nucleic acids. *Methods Mol. Biol.* **313**, 15–20 (2006).
2. Chen, S., Zhou, Y., Chen, Y. & Gu, J. fastp: an ultra-fast all-in-one FASTQ preprocessor. *Bioinformatics* **34**, i884–i890 (2018).
3. Li, H. & Durbin, R. Fast and accurate short read alignment with Burrows-Wheeler transform. *Bioinformatics* **25**, 1754–1760 (2009).
4. Li, H. *et al.* The Sequence Alignment/Map format and SAMtools. *Bioinformatics* **25**, 2078–2079 (2009).
5. Van der Auwera, G. A. *et al.* From fastQ data to high-confidence variant calls: The genome analysis toolkit best practices pipeline. *Curr. Protoc. Bioinforma.* **43**, 11.10.1-11.10.33 (2013).
6. Quinlan, A. R. & Hall, I. M. BEDTools: a flexible suite of utilities for comparing genomic features. *Bioinformatics* **26**, 841–842 (2010).
7. Thorvaldsdottir, H., Robinson, J. T. & Mesirov, J. P. Integrative Genomics Viewer (IGV): high-performance genomics data visualization and exploration. *Brief. Bioinform.* **14**, 178–192 (2013).
8. Poplin, R. *et al.* Scaling accurate genetic variant discovery to tens of thousands of samples. *bioRxiv* 201178 (2018) doi:10.1101/201178.
9. Fay, J. C. *et al.* A polyploid admixed origin of beer yeasts derived from European and Asian wine populations. *PLOS Biol.* **17**, e3000147 (2019).
10. Large, C. R. L. *et al.* Genomic stability and adaptation of beer brewing yeasts during serial repitching in the brewery. *bioRxiv* 2020.06.26.166157 (2020) doi:10.1101/2020.06.26.166157.
11. Imre, A. *et al.* Heme Oxygenase-1 (*HMX1*) Loss of Function increases the in-host fitness of the *Saccharomyces 'boulardii'* probiotic yeast in a mouse fungemia model. *J. Fungi* **8**, 522 (2022).
12. Rácz, H. V. *et al.* How to characterize a strain? Clonal heterogeneity in industrial *Saccharomyces* influences both phenotypes and heterogeneity in phenotypes. *Yeast* **38**, 453–470 (2021).
13. Prjibelski, A., Antipov, D., Meleshko, D., Lapidus, A. & Korobeynikov, A. Using SPAdes de novo assembler. *Curr. Protoc. Bioinforma.* **70**, e102 (2020).
14. Prjibelski Cabanettes, F., Klopp, C. D-GENIES: dot plot large genomes in an interactive, efficient and simple way. *PeerJ* **6**, e4958 (2018).
15. Bolyen, E. *et al.* Reproducible, interactive, scalable and extensible microbiome data science using QIIME 2. *Nat. Biotechnol.* **37**, 852–857 (2019).
16. Callahan, B. J. *et al.* DADA2: High-resolution sample inference from Illumina amplicon data. *Nat. Methods* **13**, 581–583 (2016).
17. Rognes, T., Flouri, T., Nichols, B., Quince, C. & Mahé, F. VSEARCH: a versatile open source tool for metagenomics. *PeerJ* **4,** e2584 (2016).
18. Lu, Y. *et al.* MicrobiomeAnalyst 2.0: comprehensive statistical, functional and integrative analysis of microbiome data. *Nucleic Acids Res.* **51**, W310–W318 (2023).
