# Supplementary material for: ENA1 deficiency attenuates Saccharomyces ‘boulardii’ probiotic yeast virulence in immunosuppressed mouse fungaemia model
Source: Commun Biol. 2026 Mar 6;9:542. doi: 10.1038/s42003-026-09763-z (PMC13096209; doi:10.1038/s42003-026-09763-z)
Supplement: Supplementary file 2 — Description of Additional Supplementary Files [file 42003_2026_9763_MOESM2_ESM.pdf]

# Description of Additional Supplementary Files

**File name:** Supplementary Data
**Description:** The source data behind the graphs and figures in the paper.
