# Supplementary material for: ENA1 deficiency attenuates Saccharomyces ‘boulardii’ probiotic yeast virulence in immunosuppressed mouse fungaemia model
Source: Commun Biol. 2026 Mar 6;9:542. doi: 10.1038/s42003-026-09763-z (PMC13096209; doi:10.1038/s42003-026-09763-z)
Supplement: Supplementary file 4 — Reporting Summary [file 42003_2026_9763_MOESM4_ESM.pdf]

nature portfolio

Corresponding author(s): Walter P. Pfliegler

Last updated by author(s): Feb 16, 2026

Reporting Summary

Nature Portfolio wishes to improve the reproducibility of the work that we publish. This form provides structure for consistency and transparency in reporting. For further information on Nature Portfolio policies, see our Editorial Policies and the Editorial Policy Checklist.

Statistics

For all statistical analyses, confirm that the following items are present in the figure legend, table legend, main text, or Methods section.

n/a	Confirmed	
☐	☒	The exact sample size (*n*) for each experimental group/condition, given as a discrete number and unit of measurement
☐	☒	A statement on whether measurements were taken from distinct samples or whether the same sample was measured repeatedly
☐	☒	The statistical test(s) used AND whether they are one- or two-sided<br>*Only common tests should be described solely by name; describe more complex techniques in the Methods section.*
☒	☐	A description of all covariates tested
☐	☒	A description of any assumptions or corrections, such as tests of normality and adjustment for multiple comparisons
☐	☒	A full description of the statistical parameters including central tendency (e.g. means) or other basic estimates (e.g. regression coefficient) AND variation (e.g. standard deviation) or associated estimates of uncertainty (e.g. confidence intervals)
☐	☒	For null hypothesis testing, the test statistic (e.g. *F*, *t*, *r*) with confidence intervals, effect sizes, degrees of freedom and *P* value noted<br>*Give P values as exact values whenever suitable.*
☒	☐	For Bayesian analysis, information on the choice of priors and Markov chain Monte Carlo settings
☒	☐	For hierarchical and complex designs, identification of the appropriate level for tests and full reporting of outcomes
☒	☐	Estimates of effect sizes (e.g. Cohen's *d*, Pearson's *r*), indicating how they were calculated

Our web collection on statistics for biologists contains articles on many of the points above.

Software and code

Policy information about availability of computer code

Data collection	Software was used for data analysis, not for collection.
Data analysis	Genomics: Mapping to the PY0001 reference genome (accession no. ASM2473226v1) was performed using the mem option of BWA 0.7.17. Sorted BAM files were obtained using Samtools 1.7.4 and Picard-tools 2.23.8. was used to mark duplicated reads. We used BEDTools 2.30.06 to calculate median coverage. Using BAM files, local realignment around indels and joint variant calling and filtering for the strains and isolates were performed with GATK 4.1.9.0.5. For a draft assembly of the LecC-integrated PY0001 strain, we used Spades 3.12.013 with default settings on the fastp-trimmed Illumina reads.

For manuscripts utilizing custom algorithms or software that are central to the research but not yet described in published literature, software must be made available to editors and reviewers. We strongly encourage code deposition in a community repository (e.g. GitHub). See the Nature Portfolio guidelines for submitting code & software for further information.

Data

Policy information about availability of data

All manuscripts must include a data availability statement. This statement should provide the following information, where applicable:

- Accession codes, unique identifiers, or web links for publicly available datasets
- A description of any restrictions on data availability
- For clinical datasets or third party data, please ensure that the statement adheres to our policy

Raw sequencing reads used in this study are deposited in NCBI SRA under BioProject PRJNA1165191 and PRJNA1358987. Cohort-called variant files are deposited in FigShare (doi: 10.6084/m9.figshare.27105919). Raw data for each graph is included in Supplementary Data File.

Research involving human participants, their data, or biological material

Policy information about studies with human participants or human data. See also policy information about sex, gender (identity/presentation), and sexual orientation and race, ethnicity and racism.

Reporting on sex and gender	No human participants.
Reporting on race, ethnicity, or other socially relevant groupings	No human participants.
Population characteristics	No human participants.
Recruitment	No human participants.
Ethics oversight	Patient data about the isolation source of the clinical isolates was available, but not directly collected for this study. All probiotic clinical isolates from the hospital's collection were included in the study, but patient data was not relevant for the current study. Patient data were handled in accordance with EU, state, and local regulations with a clinical study ethics approval from the Regional and Institutional Research Ethics Council of Debrecen (DE RKEB/IKEB 5194-2019).

Note that full information on the approval of the study protocol must also be provided in the manuscript.

Field-specific reporting

Please select the one below that is the best fit for your research. If you are not sure, read the appropriate sections before making your selection.

☒ Life sciences ☐ Behavioural & social sciences ☐ Ecological, evolutionary & environmental sciences

For a reference copy of the document with all sections, see nature.com/documents/nr-reporting-summary-flat.pdf

Life sciences study design

All studies must disclose on these points even when the disclosure is negative.

Sample size	In this study four commercial isolates of the S. 'boulardii' were used, originating from two batches each of two different probiotic supplements. Ten clinical yeast isolates were used, this represented each and every probiotic isolate available in our collection from the university clinics of Debrecen and Szeged in Hungary during the time this study was conducted. Gene deletions were performed using CRISPR/Cas9 genome editing in the case of two commercial and four clinical yeast isolates to represent both commercial isolates and clinical isolates in the study, enabling us to compare slightly different genetic backgrounds.
Data exclusions	No obtained data was ecluded from the results.
Replication	Phenotypic measurements were done on 3 replicate samples each, mouse experiments were carried out with 7–9 artifically infected mouse specimens per yeast isolate and 8 mouse specimens per gavaging experiment.
Randomization	Not relevant, the main focus was the yeast strains, for mouse infection experiments, inoculated mice were chosen randomly.
Blinding	Blinding was not relevant, experiments were carried out with microbes.

Reporting for specific materials, systems and methods

We require information from authors about some types of materials, experimental systems and methods used in many studies. Here, indicate whether each material, system or method listed is relevant to your study. If you are not sure if a list item applies to your research, read the appropriate section before selecting a response.

Materials & experimental systems

n/a	Involved in the study	
☒	☐	Antibodies
☒	☐	Eukaryotic cell lines
☒	☐	Palaeontology and archaeology
☐	☒	Animals and other organisms
☐	☒	Clinical data
☒	☐	Dual use research of concern
☒	☐	Plants

Methods

n/a	Involved in the study	
☒	☐	ChIP-seq
☒	☐	Flow cytometry
☒	☐	MRI-based neuroimaging

Animals and other research organisms

Policy information about studies involving animals; ARRIVE guidelines recommended for reporting animal research, and Sex and Gender in Research

Laboratory animals	For the kidney burden experiments BALB/c immunocompromised female mice (n= 7–9 per isolate; 21–23 g body weight; Charles River) were used.). To test viability and clearance in the gut, 8-week-old female C57BL/6J mice (n=24 total, n=8 per group) with normal microbiome (i.e. neither germ-free, nor specific-pathogen-free animals) and immune status were used.
Wild animals	None.
Reporting on sex	Only female mice were used for the experiments, following typical research setups in medical mycology infection experiments.
Field-collected samples	None.
Ethics oversight	The experiments were approved by the Animal Care Committee of the University of Debrecen, Debrecen, Hungary (permission no. 12/2014 DEMÁB). The gavaging experiment was approved by the NC State University Institutional Animal Care and Use Committee (IACUC; protocol ID: 23-434).

Note that full information on the approval of the study protocol must also be provided in the manuscript.

Clinical data

Policy information about clinical studies

All manuscripts should comply with the ICMJE guidelines for publication of clinical research and a completed CONSORT checklist must be included with all submissions.

Clinical trial registration	This is not a clinical trial, hence registration is not applicable. Only the microbial isolate collection of two clinics was involved.
Study protocol	Not relevant, the work concerns microbes from isolate collections of two clinics, obtained prior to and independent of the current study.
Data collection	Patient data were handled in accordance with EU, state, and local regulations with a clinical study ethics approval from the Regional and Institutional Research Ethics Council of Debrecen (DE RKEB/IKEB 5194-2019). Data was not collected for this study, merely was available as accompanying data for the isolates in the clinical microbial isolate collections.
Outcomes	Not relevant, the work concerns microbes from an isolate collection of two clinics, obtained prior to and independent of the current study.

Plants

Seed stocks	Not relevant.
Novel plant genotypes	Not relevant.
Authentication	Not relevant.
